# Whi2 is a conserved negative regulator of TORC1 in response to low amino acids

**DOI:** 10.1371/journal.pgen.1007592

**Published:** 2018-08-24

**Authors:** Xianghui Chen, Guiqin Wang, Yu Zhang, Margaret Dayhoff-Brannigan, Nicola L. Diny, Mingjun Zhao, Ge He, Cierra N. Sing, Kyle A. Metz, Zachary D. Stolp, Abdel Aouacheria, Wen-Chih Cheng, J. Marie Hardwick, Xinchen Teng

**Affiliations:** 1 Jiangsu Key Laboratory of Neuropsychiatric Diseases and College of Pharmaceutical Sciences, Soochow University, Suzhou, Jiangsu, China; 2 W. Harry Feinstone Department of Molecular Microbiology and Immunology, Johns Hopkins University Bloomberg School of Public Health, Baltimore, MD, United States of America; 3 ISEM, Institut des Sciences de l’Evolution de Montpellier, Université de Montpellier, CNRS, EPHE, IRD, Montpellier, France; 4 Department of Pharmacology and Molecular Sciences, Johns Hopkins University School of Medicine, Baltimore, MD, United States of America; Sheffield University, UNITED KINGDOM

## Abstract

Yeast *WHI2* was originally identified in a genetic screen for regulators of cell cycle arrest and later suggested to function in general stress responses. However, the function of Whi2 is unknown. Whi2 has predicted structure and sequence similarity to human KCTD family proteins, which have been implicated in several cancers and are causally associated with neurological disorders but are largely uncharacterized. The identification of conserved functions between these yeast and human proteins may provide insight into disease mechanisms. We report that yeast *WHI2* is a new negative regulator of TORC1 required to suppress TORC1 activity and cell growth specifically in response to low amino acids. In contrast to current opinion, *WHI2* is dispensable for TORC1 inhibition in low glucose. The only widely conserved mechanism that actively suppresses both yeast and mammalian TORC1 specifically in response to low amino acids is the conserved SEACIT/GATOR1 complex that inactivates the TORC1-activating RAG-like GTPases. Unexpectedly, Whi2 acts independently and simultaneously with these established GATOR1-like Npr2-Npr3-Iml1 and RAG-like Gtr1-Gtr2 complexes, and also acts independently of the PKA pathway. Instead, Whi2 inhibits TORC1 activity through its binding partners, protein phosphatases Psr1 and Psr2, which were previously thought to only regulate amino acid levels downstream of TORC1. Furthermore, the ability to suppress TORC1 is conserved in the SKP1/BTB/POZ domain-containing, Whi2-like human protein KCTD11 but not other KCTD family members tested.

## Author summary

Yeast and human cells respond to declining levels of available nutrients to prepare ahead for leaner times. The detailed mechanisms of nutrient sensing are not well understood, but defects in these processes have key roles in diseases such as cancer. The evolutionarily conserved protein complex TORC1 is the control hub for responding to both high and low nutrients, particularly amino acids. We identified yeast Whi2 and the human tumor suppressor KCTD11 as novel suppressors of TORC1 activity in low amino acid conditions, and we investigated the detailed mechanisms for Whi2. Unexpectedly, Whi2 works differently from the usual mechanism where TORC1 is controlled by the SEACIT-Gtr complex (mammalian GATOR1-RAG complex). Furthermore, both the Whi2 and the SEACIT-Gtr pathways work independently and together in parallel to suppress TORC1. For this function, Whi2 requires its binding partners, the yeast protein phosphatases Psr1 and Psr2, which were previously thought to function downstream of TORC1 in amino acid signaling. These studies have important implications for human KCTD11 to help advance the understanding of its pathological role.

## Introduction

The understudied Whi2 protein of *Saccharomyces cerevisiae* and its fungal homologs share predicted domain structure and sequence similarity with the family of human KCTD proteins [1] (S1 Fig). Despite primary sequence divergences, a homologous SKP1/BTB/POZ domain is identifiable (IPR011333) in the N-terminal portion of both protein types. KCTD family members have been associated with several types of cancer, epilepsy and other disorders [2–5]. However, the functions of these proteins in any species are not understood and the mechanisms of disease caused by KCTD mutations are unknown.

Early studies revealed that yeast Whi2 is required to halt the cell cycle, to suppress cyclin expression, and for entry into stationary phase [6–9]. Another study found that Whi2 is important for handling general environmental stresses by interacting with the protein phosphatase Psr1, which has been suggested to dephosphorylate and activate the general stress response transcription factor Msn2 [10]. More recently, Psr1 and Psr2 were reported to inhibit plasma membrane transporter activity in response to TORC1 inhibition [11]. However, Whi2, Psr1 and Psr2 were not previously reported to function as upstream regulators of TORC1.

Diverse functions of mammalian KCTD family proteins have been reported, including KCTD13 and TNFAIP1 binding to Rho GTPases Rnd2/3 during brain development [12], KCTD10 binding to PCNA in DNA repair [13], *Drosophila* Insomniac (KCTD5) interacting with cullin 3 in sleep regulation [14], and KCTD8, KCTD12, and KCTD16 interacting with GABA_B_ G-protein coupled receptors in neurons [15]. In addition, mouse *Kctd11* was identified as a tumor suppressor gene near *TP53* in an *in vivo* mouse screen [16], and human *KCTD11* deficiency is suggested to contribute to several cancers including medulloblastoma [2], hepatocellular carcinoma [17], and prostate adenocarcinoma [18].

We identified yeast *WHI2* by sequencing the genomes of several knockout strains of the mitochondrial fission factor Fis1 to identify the spontaneous mutations responsible for a robust growth phenotype. Remarkably, three independent *Δfis1* strains each had a different premature stop codon in *WHI2* [19]. Spontaneous *WHI2* mutations are not responsible for the mitochondrial fission defects due to *FIS1* deletion, but are responsible for two seemingly contradictory phenotypes. *WHI2*-deficiency causes sensitivity to multiple stresses, including heat, acetic acid, killer viruses and ROS/H_2_O_2_, and paradoxically also causes excessive overgrowth compared to wild type in media containing reduced amino acid levels [1, 19–21], Our findings are consistent with previous cell cycle and cell stress studies [6–9, 22].

We also identified yeast *WHI2* in a genome-wide screen for genes required to suppress cell growth/division under low amino acid conditions [1]. This screen also identified the deletion strains of *NPR2 and NPR3/RMD11* [1], which are components of the conserved protein complex SEACIT (human GATOR1) known to actively suppress TORC1 kinase activity and cell growth when the availability of environmental amino acids declines [23, 24]. Under low amino acid conditions, the conserved GTPase activating (GAP) function of SEACIT (human GATOR1) inhibits the TORC1-activating RAG-like GTPase Gtr1 (human RAGA/B) [25, 26]. Additional negative regulators of mTORC1 in this same amino acid signaling pathway have been identified in mammals, such as Sestrin2 [27], the KICSTOR complex [28] and CASTOR1/2 [29], but lack obvious orthologs in fungi with rare exception [30]. An alternative negative regulator of TORC1/mTORC1 in some yeast species and in mammals is the TSC complex, a GAP for the TORC1-activating GTPase Rheb [31], but TSC1/2 are not found in *Saccharomyces cerevisiae*. Therefore, we investigated a potential role for Whi2 in this SEACIT–Gtr1/Gtr2–TORC1 pathway. Instead, we found that Whi2 suppresses TORC1 independently of the SEACIT-Gtr pathway and independently of the PKA pathway. However, both Whi2 and SEACIT-Gtr can work in concert to suppress cell growth specifically in response to low amino acids. Although the detailed molecular mechanisms are not known, we show that Whi2 suppresses TORC1 activity through its binding partners, protein phosphatases Psr1 and Psr2, revealing a new role for Psr1/Psr2 upstream of TORC1. Furthermore, both exogenous and endogenous human KCTD11, a Whi2-like protein harboring a homologous SKP1/BTB/POZ domain, suppress mTORC1 activity in mammalian cells in response to low amino acids, indicating an evolutionarily conserved function.

## Results

### *WHI2* is required to suppress TORC1 activity in low amino acids

**Fig 1:**
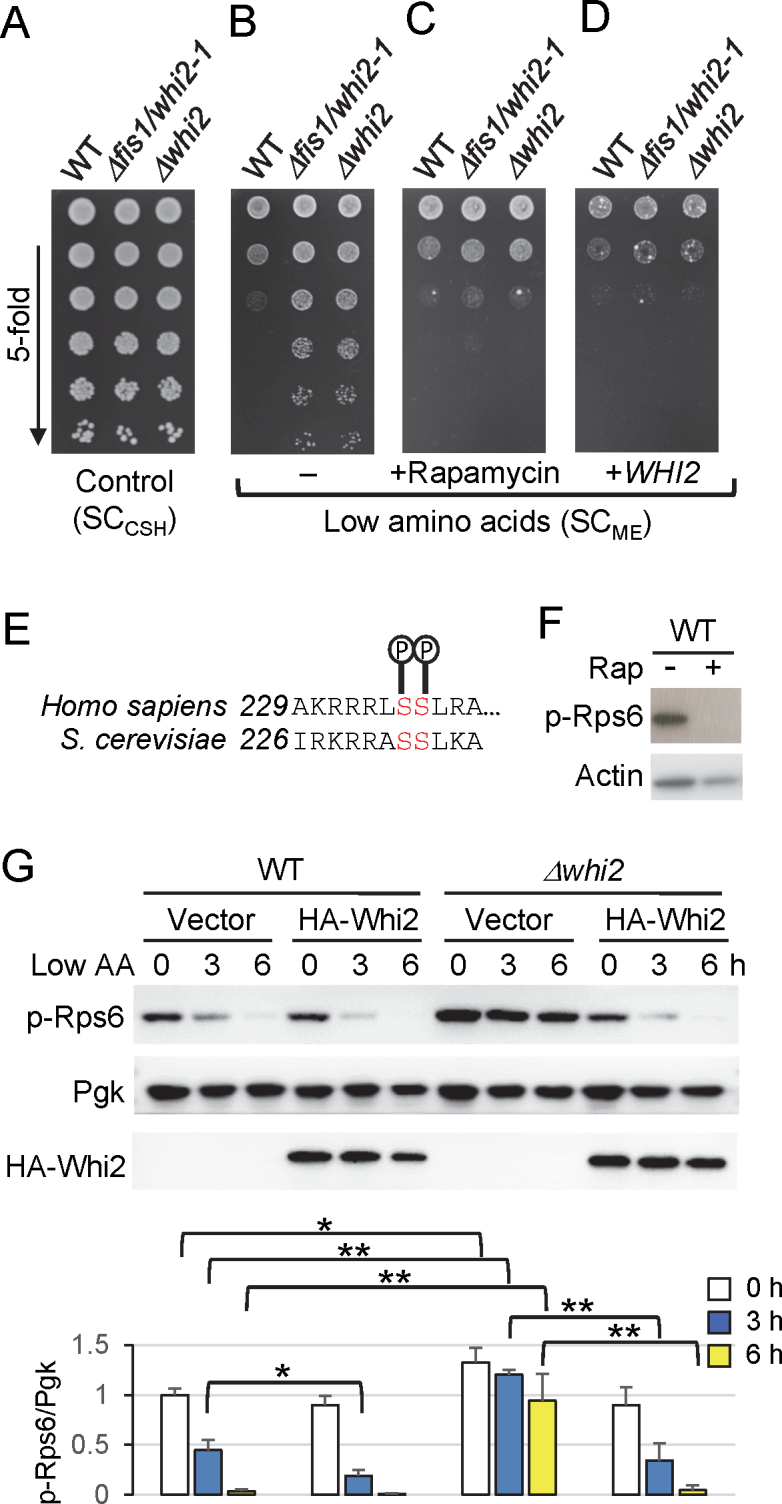
Whi2 is required to suppress TORC1 activity in low amino acids. (A) Density-matched liquid yeast cultures grown in YPD were spotted on SC_CSH_ (1,762 mg/L amino acids) agar plates in 5-fold serial dilutions (starting with undiluted samples) and incubated for 2 days. (B) The same cultures from panel A were spotted on SC_ME_ (1,200 mg/L amino acids) in the same manner and incubated for 3 days. (C) The same cultures from panel A were spotted on SC_ME_ containing 2.5 ng/mL rapamycin (Sigma). (D) Parallel cultures of yeast strains transformed with a *WHI2* (CEN-URA) expression plasmid containing a genomic DNA fragment of the *WHI2* ORF and its native regulatory sequences (Table 2) were analyzed as in panel B. Images in panels A-D were adjusted equally to reflect original results. Representative of many independent experiments are shown. (E) Amino acid sequence alignment of conserved phosphorylation sites near or at the C-terminus of human and yeast ribosomal protein S6, respectively. (F) Immunoblot of whole cell lysates prepared from wild type yeast treated with/without 200 nM rapamycin for 1 h in YPD and analyzed using anti-mammalian phospho-S6 (Ser235/S236) and actin antibodies. (G) TORC1 activity after switching from control/high (SC_CSH_) to low amino acid media (SC_ME_) using anti-phospho-Rps6 immunoblots of lysates from strains transformed with empty vector or a constitutive *PGK1* promoter-driven N-terminal HA-tagged Whi2 expression vector. Equal loading of samples was achieved primarily by using density-matched cultures and monitored with anti-Pgk. Corresponding bars below show quantification of TORC1 activity (the ratio of phospho-Rps6/Pgk). Values were normalized to the average value of WT transformed with empty vector at time zero, and presented in the bar graph as means ± SD (n = 4 independent experiments). **P < 0.01; *P < 0.05; using a two-tailed T test.

We previously reported that wild type and *whi2* mutant strains of *Saccharomyces cerevisiae* grow similarly on synthetic medium containing high amino acid levels (SC_CSH_) (Fig 1A) [19]. However, on medium with ~30% lower total amino acids (SC_ME_), *whi2* mutants grow significantly more robustly than wild type BY4741 (Fig 1B) [19]. Thus, under these relatively small changes in nutrient levels compared to other studies, wild type is apparently capable of sensing the reduced amino acid levels and limiting its growth, in contrast to strains with an engineered *WHI2* gene deletion (*Δwhi2*) or a spontaneous mutation in *WHI2* (*Δfis1*/*whi2-1*, E153X) [1, 19]. These findings are consistent with original studies in late-stage cultures showing that *WHI2*-deficiency causes a failure to enter quiescence as overall nutrient levels decline [6–9]. To determine if TORC1, the major regulator of cellular responses to amino acid availability in yeast and mammals [25], is responsible for this overgrowth by *whi2* mutants, a low concentration of the TORC1 inhibitor rapamycin (2.5 ng/mL) was added to the solid plate medium. Rapamycin reduced the growth of *whi2* mutants to wild type levels (Fig 1C), similar to the effects of reintroducing *WHI2* under its native regulatory sequences on a plasmid (without rapamycin) (Fig 1D), confirming involvement of *WHI2*. Thus, *WHI2* appears to be required to restrict TORC1-dependent cell growth/division when amino acid levels dwindle and before supplies are exhausted.

To further investigate the role of *WHI2* in suppressing TORC1, we monitored TORC1 activity based on the phosphorylation status of endogenous 40S ribosomal subunit S6 (Rps6) at Ser232/Ser233 without requiring reporters or gel-shift assays. Rps6 is phosphorylated by Ypk3, which is believed to be a direct target of TORC1 in response to amino acids and nitrogen availability [32–34]. Antibodies directed against these same sites in mammalian S6 (Ser235/Ser236), which are also phosphorylated downstream of mammalian mTORC1 in response to amino acids [35, 36], readily detected phosphorylated yeast Rps6 in a TORC1-dependent manner based on inhibition by rapamycin (Fig 1E and 1F). As expected, TORC1 activity assessed by Rps6 phosphorylation is significantly reduced in wild type cells by 3 h and nearly abolished at 6 h after switching from high (SC_CSH_) to low amino acids (SC_ME_) (Fig 1G). In contrast, the *whi2* knockout has sustained TORC1 activity with only partial diminution by 6 h after media switch. Although the absolute levels of TORC1 activity can shift between independent experiments, the relationship between wild type and the *whi2* knockout is highly consistent at each time point, including at baseline (Fig 1G). Furthermore, the ability to suppress TORC1 was restored in *Δwhi2* by constitutively expressing HA-Whi2 on a plasmid, and protein expression was verified on anti-HA immunoblots (Fig 1G).

### TORC1 inhibition in low glucose does not require Whi2

**Fig 2:**
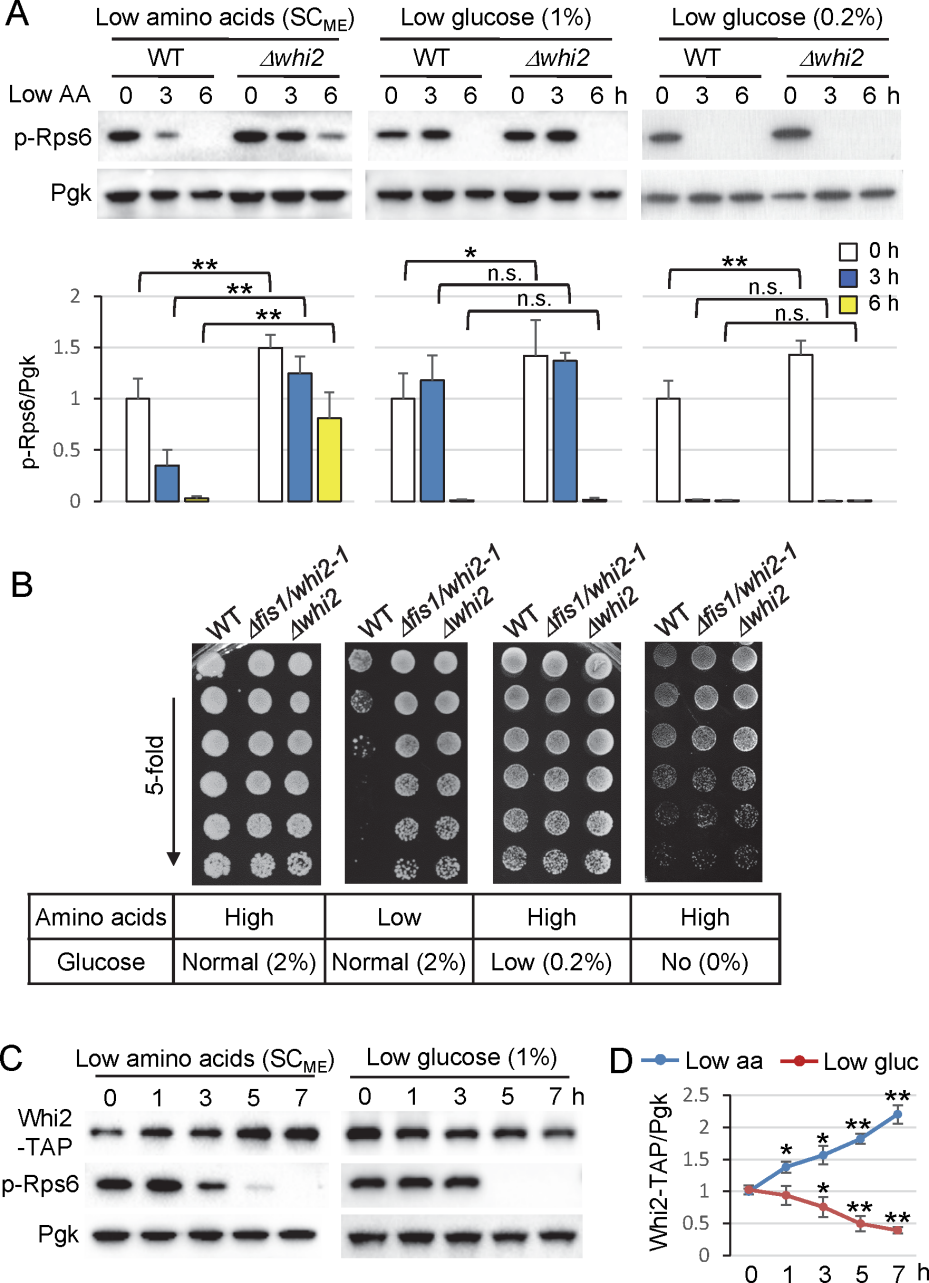
Whi2 protein levels rapidly respond to low amino acids but not low glucose. (A) Immunoblot analysis for TORC1 activity assessed with anti-phospho-RpsS6 before and after switching wild type and Δ*whi2* from control/high (SC_CSH_) to low amino acids (SC_ME_), and from normal (SC_CSH_, 2% glucose) to low glucose (SC_CSH_, 1% glucose) and measuring TORC1 activity as in Fig 1G. Equal loading was achieved primarily by using density-matched cultures and monitored with anti-Pgk. Corresponding bars below show quantification of TORC1 activity (the ratio of phospho-Rps6/Pgk). Values were normalized to the average value of WT at time zero, and presented in the bar graph as means ± SD (n = 4, 4, and 3 independent experiments for each condition respectively). **P < 0.01; *P < 0.05; n.s., not significant, compared to the respective WT control using a two-tailed T test. (B) Growth of indicated yeast cultures spotted in 5-fold serial on control medium containing high amino acids and normal (2%) glucose (SC_CSH_), low amino acid medium (SC_ME_) with normal (2%) glucose, or SC_CSH_ containing high amino acids and very low glucose (0.2%) or no glucose (0%). All the cultures were grown in control medium containing high amino acids and normal (2%) glucose (SC_CSH_) overnight, then density-matched cultures were diluted in water and spotted on indicated plates. (C) Expression levels of genomic C-terminal TAP-tagged Whi2 and corresponding phospho-Rps6 levels were monitored on immunoblots before and after media changes as in panel A. (D) Quantification of expression levels of genomic C-terminal TAP-tagged Whi2 from C. Values at each time point were normalized to the average value at time zero, and presented in the graph as means ± SD (n = 3 independent experiments for low amino acid treatment, and n = 4 independent experiments for low glucose treatment). **P < 0.01; *P < 0.05; compared to the time zero control using a two-tailed T test.

Yeast TORC1 and its mammalian counterpart mTORC1 are particularly responsive to amino acid levels, but have also been reported to respond to low glucose in an Snf1/AMPK-dependent manner [37–40]. However, we found that TORC1 activity (phospho-Rps6) was suppressed normally in *∆whi2* after switching from standard 2% glucose to 1% or 0.2% glucose (in high amino acid SC_CSH_) (Fig 2A). Overall colony growth density (colony size) was detectably reduced on low glucose and obviously reduced without glucose supplementation, but independently of *WHI2* (Fig 2B). These findings are in contrast to other studies identifying *WHI2* as a general stress response gene, including for low glucose conditions [10, 41].

**Fig 3:**
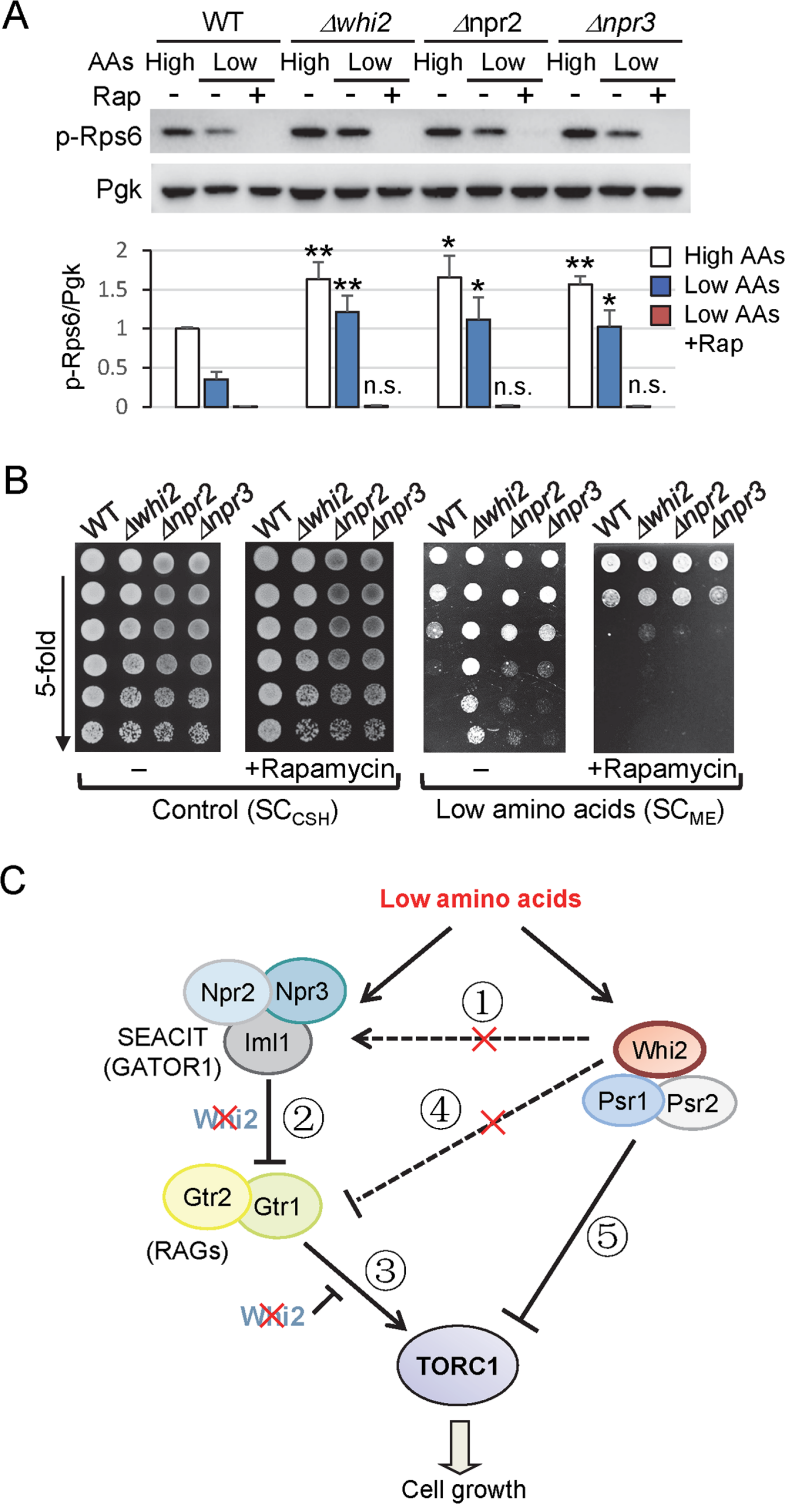
Whi2 is a potent suppressor of TORC1 similar to Npr2 and Npr3. (A) Immunoblot analysis detecting sustained TORC1 activity in mutant strains was assessed with anti-phospho-Rps6 before and after switching from control medium (SC_CSH_) to low (L) amino acids (SC_ME_) for 3 h with/without addition of 20 ng/mL rapamycin for an additional 30 min (Rap). Equal loading was achieved primarily by optical density-matched cultures and monitored with anti-Pgk. Corresponding bars below show quantification of TORC1 activity (the ratio of phospho-Rps6/Pgk). Values were normalized to the average value of WT before switching media, and presented in the bar graph as means ± SD (n = 4 independent experiments). **P < 0.01; *P < 0.05; n.s., not significant, compared to the respective WT control using a two-tailed T test. (B) Growth of density-matched cultures spotted in parallel on control/high levels of amino acids (SC_CSH_) without or with 2.5 ng/mL rapamycin, and on low amino acid levels (SC_ME_) without or with 2.5 ng/mL rapamycin. Representative of 3 independent experiments are shown. (C) Model depicting the position of Whi2 relative to the Iml1-Npr2-Npr3 and Gtr1-Gtr2 axis in the yeast TORC1 pathway. See text for explanation of numbered steps.

If *WHI2* has a specific role in communicating low amino acid status, then it is expected that protein expression levels of Whi2 may be sustained or induced even in low amino acid conditions. Consistent with a role in responding to low amino acids, but not to low glucose, endogenous Whi2 protein levels (detected with a knockin C-terminal TAP-tag [42]) are consistently increased by 1 h after switching to low amino acids (SC_ME_ 2% glucose), but decreased by 1–2 h in low glucose (SC_CSH_ 1% glucose) (Fig 2C). These inverse trajectories of Whi2 protein levels continued over the 7 h time course (Fig 2D) despite equivalent shut-off of TORC1 activity (phospho-Rps6) in both low amino acids and low glucose conditions (Fig 2C). Notably, induction of Whi2 protein reproducibly precedes TORC1 suppression, consistent with a causal role for Whi2. Furthermore, the sustained phospho-Rps6 status observed in *whi2* mutants grown in low amino acids is abolished by a 30 min treatment with TORC1 inhibitor rapamycin (20 ng/mL), verifying a role for TORC1 (Fig 3A). These findings are consistent with the model that Whi2 responds specifically to low amino acid levels by restricting TORC1 activity. Therefore, we further investigated a potential role for Whi2 in known amino acid signaling pathways to TORC1 [24, 43].

### Whi2 regulates TORC1 in parallel with Npr2-Npr3-Iml1 (SEACIT/GATOR1)

*WHI2* was the top hit in our genome-wide screen of 4,847 gene deletion strains for overgrowth on low amino acids (SC_ME_) [1]. This same screen also identified *NPR2* and *NPR3* [1], components of the TORC1-suppressing SEACIT complex (mammalian GATOR1). Several other hits in this screen were false positives due to spontaneous *WHI2* mutations [1, 19]. However, backcrosses and tetrad analysis revealed no secondary mutations to explain the phenotypes of *Δnpr2* and *Δnpr3* (S2 Fig). *NPR2* and *NPR3* were first linked to the TORC1 pathway when they were identified as the top hits in a nitrogen starvation reporter screen while in search of genes that communicate amino acid depletion to TORC1 [43]. Yeast Npr2 and Npr3 together with their catalytic subunit Iml1 form the SEACIT complex and, like their mammalian counterparts NPRL2, NPRL3 and DEPDC5 in the GATOR1 complex [23], negatively regulate TORC1 in response to low amino acids [24]. Therefore, we directly compared *∆npr2* and *∆npr3* to *Δwhi2* for dysregulation of TORC1 activity in our mild amino acid depletion assay.

Similar to *Δwhi2*, both *Δnpr2* and *Δnpr3* strains have sustained phosphorylation of Rps6 at 3 h after switching to low amino acids (SC_ME_), which is dependent on TORC1 based on sensitivity to rapamycin (20 ng/mL, 30 min) (Fig 3A), consistent with the original study [43]. Sustained TORC1 activity is consistent with sustained cell growth on low amino acid plates (SC_ME_) as both *∆npr2* and *Δnpr3* exhibit strong overgrowth (albeit reduced compared to *Δwhi2*) that reverts to wild type levels with rapamycin treatment (2.5 ng/mL) (Fig 3B). This low concentration of rapamycin does not cause general growth inhibition of tested strains on rich media containing high amino acids (Fig 3B). Thus, Whi2 appears to be a potent suppressor of TORC1 and cell growth in response to specific signals (Fig 3C).

Two additional readouts for TORC1 activity were used to further confirm that Whi2 suppresses TORC1. The transcription of *DAL80* mRNA is known to be downregulated when TORC1 is active and upregulated when TORC1 is inactive [44]. Using a *DAL80* promoter-driven GFP reporter plasmid to monitor the expression of *DAL80* [43], we observed significantly reduced *prDAL80*-GFP levels in *Δwhi2*, *Δnpr2* and *Δnpr3* compared to wild type, indicating higher TORC1 activity in the mutants (S3A Fig). We also tested the phosphorylation status of Npr1, suggested to be a direct target of TORC1 [45]. Based on the characteristic up-shifted migration of phosphorylated Npr1 on immunoblots, Npr1 is hyperphosphorylated in *Δwhi2* and partially hyperphosphorylated in *Δnpr2* compared to wild type cells at time 0. Npr1 became further shifted in *Δnpr2* over 3–6 h in low amino acids, approximately co-migrating with Npr1 in *Δwhi2* (S3B Fig), consistent with hyperphosphorylation of Npr1 in low nitrogen in *Δnpr2 and* in *Δnpr3* [43]. Thus, in addition to rapamycin sensitivity, three independent readouts for TORC1 indicate that *WHI2* is required to suppress TORC1 activity in low amino acids.

**Fig 4:**
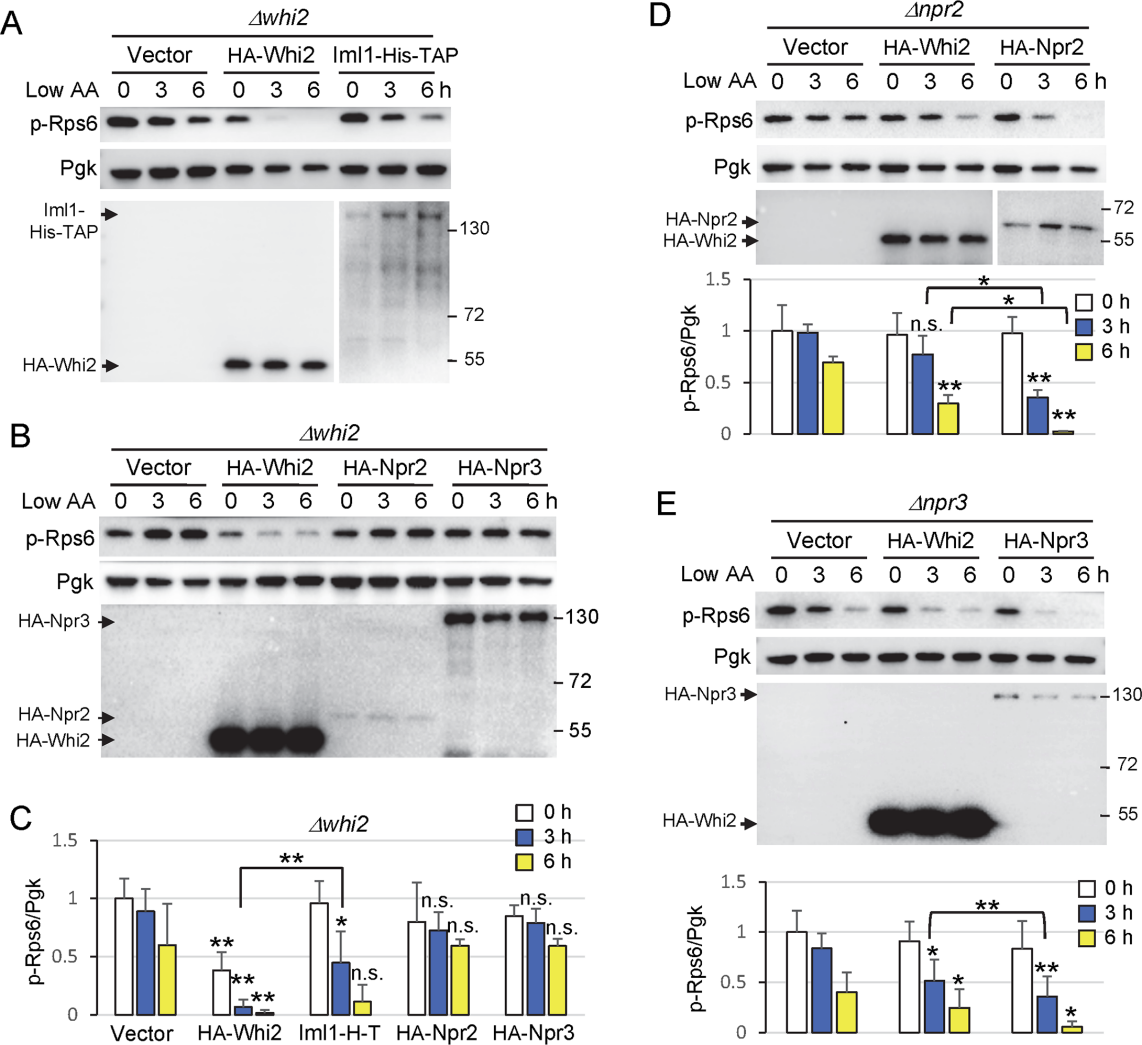
Whi2 functions parallel to the Iml1-Npr2-Npr3 complex in low amino acids. (A-B) Immunoblot analysis for TORC1 activity assessed with anti-phospho-RpsS6 in Δ*whi2* expressing the indicated proteins (second line) before and after amino acid reduction (switch from SC_CSH_ to SC_ME_). Anti-HA was used to detect N-terminal HA-tagged Whi2, Npr2, and Npr3 expressed from the *PGK1* promoter, and anti-His was used to detect C-terminal His-TAP-tagged Iml1 expressed from the constitutive *ADH1* promoter. For reproduction, contrast-adjusted images are shown for HA blots in panel B. Equal loading was achieved primarily by optical density-matched cultures and monitored with anti-Pgk. (C) Quantification of TORC1 activity (the ratio of phospho-Rps6/Pgk) from A-B. Values were normalized to the average value of empty vector at time zero, and presented in the bar graph as means ± SD (n = 3–6 independent experiments). **P < 0.01; *P < 0.05; n.s., not significant, compared to the respective vector control using a two-tailed T test. (D-E) Immunoblot analysis for TORC1 activity in *Δnpr2* and *Δnpr3* expressing the indicated proteins (second line) as described in A-B. A longer exposure of the same blot is shown for HA-Npr2 in D. Corresponding bars below show quantification of TORC1 activity as described in C. n = 3 (in D) and n = 4 (in E) independent experiments. **P < 0.01; *P < 0.05; n.s., not significant, compared to the respective vector control using a two-tailed T test.

Whi2 could potentially regulate TORC1 by several different mechanisms (Fig 3C). To determine if *WHI2* is required for the Npr2/3-containing SEACIT complex to suppress TORC1 activity, we first asked if the catalytic subunit of the SEACIT complex, Im1, which has GAP activity for Gtr1 [24], can suppress the sustained TORC1 activity in *Δwhi2*. Indeed, enforced expression of an enzymatically active Iml1 with C-terminal His-TAP-tags from a plasmid [24] suppressed phospho-Rps6 levels in *Δwhi2* following a switch to low amino acids, although less efficiently than HA-Whi2 (Fig 4A and 4C). This indicates that Whi2 is not essential for SEACIT to suppress TORC1 (② in Fig 3C), and raises the possibility that Whi2 may act in a parallel genetic pathway independently of the SEACIT complex (④ or ⑤ in Fig 3C). Although HA-Npr2 and HA-Npr3 had no effect on TORC1 activity in the absence of *WHI2*, these non-catalytic subunits, in contrast to Iml1, may be unable to enhance SEACIT function independently (Fig 4B and 4C). The failure of expressed Npr2 and Npr3 to rescue *whi2*-deficiency is not an inherent defect of these constructs, as each could fully rescue its respective deletion strain (Fig 4D and 4E). However, because we did not express all SEACIT components simultaneously, these results alone cannot exclude the possibility that Whi2 might regulate SEACIT (① in Fig 3C). To further address this point, we used the inverse approach. We found that enforced expression of HA-Whi2 suppresses overactive TORC1 (phospho-Rps6) in both *∆npr2* and *∆npr3* after switching to low amino acids, but again only partially (Fig 4D and 4E). This is evident because HA-Whi2 was consistently more effective than empty vector but also consistently less effective than re-expression of Npr2 or Npr3 in their respective knockouts by 3 h after switching to low amino acids (Fig 4D and 4E, compare to empty vector in each case). Thus, Npr2 and Npr3 are not essential for Whi2 to affect TORC1, indicating that Whi2 does not act upstream of the SEACIT complex (① in Fig 3C). Taking together the observed reciprocal partial rescues, these results are consistent with a model where Whi2 and the Npr2-Npr3-Iml1 SEACIT complex function in parallel pathways to negatively regulate TORC1.

### Whi2 modulates TORC1 activity independently of the Gtr1 (RAG) GTPase

**Fig 5:**
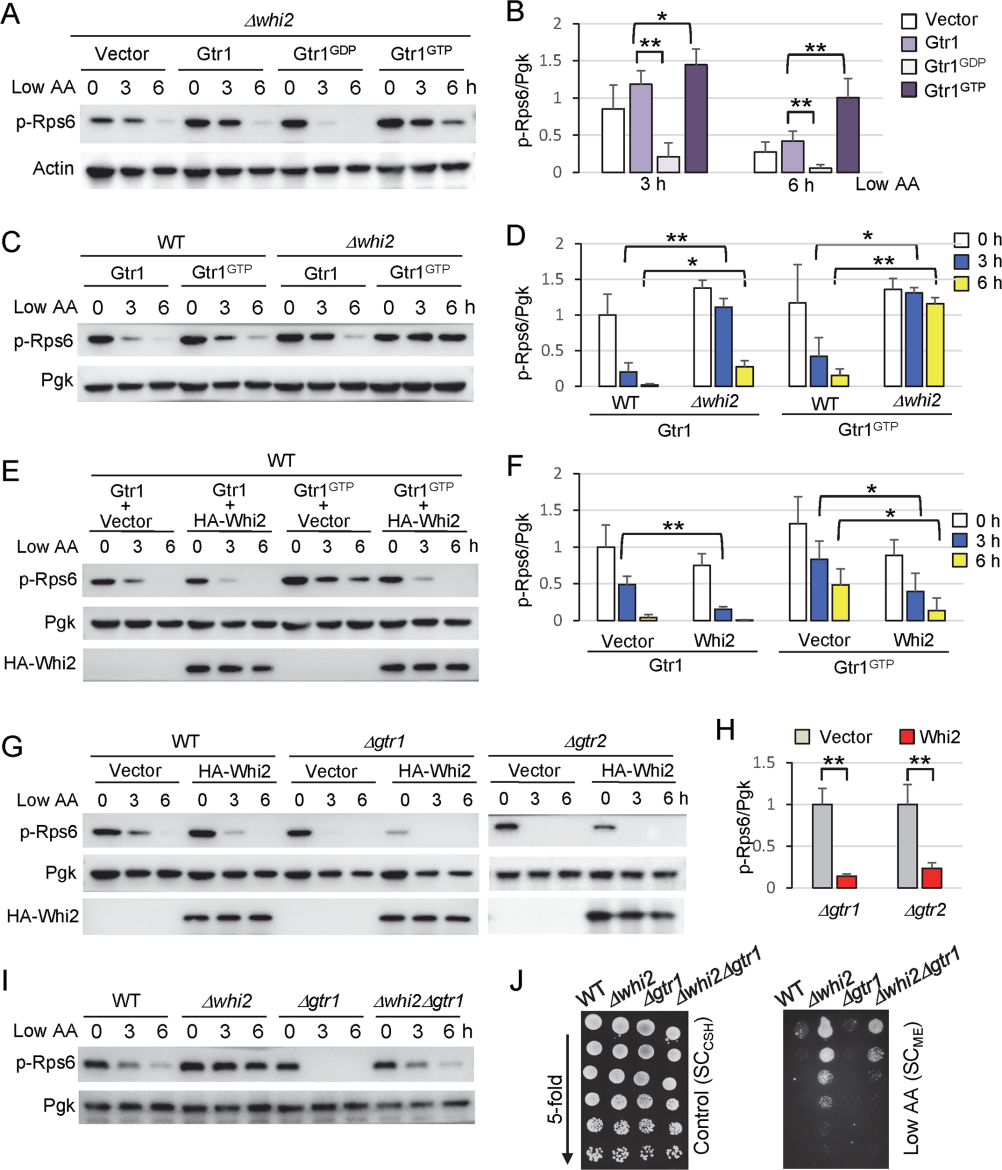
Whi2 modulates TORC1 activity independently of Gtr complex. (A, C, E, G, I) Assessment of TORC1 activity by anti-phospho-Rps6 immunoblot analysis of the indicated yeast strains at increasing times after switching from control/high SC_CSH_ to low amino acid SC_ME_ media as described in Fig 1G. Representative of >3 independent experiments is shown. (B) Quantification of TORC1 activity (ratio of phospho-Rps6/Pgk) from panel A at 3 h and 6 h after switching from control/high SC_CSH_ to low amino acid SC_ME_ media. Values were normalized to the average value of empty vector at time zero, and presented as means ± SD (n = 4 independent experiments). p = 6.1 x 10^−5^ (3h), p = 0.0073 (6h) between Gtr1 and Gtr1^GDP^. p = 0.023 (3h), p = 0.0062 (6h) between Gtr1 and Gtr1^GTP^ using paired two-tailed t-test. (D) Quantification of TORC1 activity (ratio of phospho-Rps6/Pgk) from panel C. Values were normalized to the average value of WT expressing wild type Gtr1 at time zero, and presented as means ± SD (n = 3 independent experiments). p = 7.6 x 10^−4^ (3h), p = 0.038 (6h) between WT and Δ*whi2* expressing wild type Gtr1. p = 0.037 (3h), p = 0.0077 (6h) between WT and Δ*whi2* expressing Gtr1 ^GTP^ using paired two-tailed t-test. (F) Quantification of TORC1 activity (ratio of phospho-Rps6/Pgk) from panel E. Values were normalized to the average value of WT containing wild type Gtr1 + empty vector at time zero, and presented as means ± SD (n = 4 independent experiments). p = 0.0015 (3h) between vector and Whi2 co-expressed with wild type Gtr1. p = 0.018 (3h), p = 0.017(6h) between vector and Whi2 co-expressed with Gtr1 ^GTP^ using paired two-tailed t-test. (H) Quantification of TORC1 activity (the ratio of phospho-Rps6/Pgk) of Δ*gtr1* and Δ*gtr2* from G at time zero. Values were normalized to the average value of Δ*gtr1* or Δ*gtr2* containing empty vector, and presented in the bar graph as means ± SD (n = 4 independent experiments). p = 0.00014 between vector and Whi2 in Δ*gtr1*. p = 0.0013 between vector and Whi2 in Δ*gtr2* using paired two-tailed t-test. For B, D, F and H, **P < 0.01; *P < 0.05. No data were included in >1 graph. (J) Growth of indicated yeast strains spotted in parallel in 5-fold serial dilutions on both control/high (SC_CSH_) and low amino acid plates (SC_ME_). Representative of 3 independent experiments is shown.

To further extend these studies, we investigated the relationship between Whi2 and the downstream target of SEACIT, the RAG-like GTPases Gtr1/Gtr2. To signal low amino acid status to TORC1, the Npr2-Npr3-Iml1 SEACIT complex (mammalian GATOR1) is known to inhibit the Gtr1 GTPase (mammalian RAGA/B) to suppress TORC1 activity [24]. To first determine if Whi2 is required for the RAG-like GTPases to regulate TORC1, constitutively inhibitory Gtr1^GDP^(S20L) and constitutively active Gtr1^GTP^(Q65L) [46] mutants were expressed via plasmids in yeast lacking the *WHI2* gene. Both the inhibitory and activated forms of Gtr1 were still capable of modulating TORC1 activity (phospho-Rps6) even in the absence of Whi2 through 6 h after switching to low amino acids (Fig 5A and 5B). That is, Gtr1^GDP^(S20L) dramatically decreases TORC1 activity by 3 h in low amino acids, while Gtr1^GTP^(Q65L) still maintains a high TORC1 activity at 6 h compared with wild type Gtr1 (Fig 5B). Thus, Whi2 does not appear to inhibit TORC1 downstream of the Gtr1-Gtr2 complex as Whi2 is not essential for Gtr1 to modulate TORC1 activity (③ in Fig 3C).

Conversely, Whi2 does not require Gtr1 to regulate TORC1. Endogenous Whi2 suppresses TORC1 in cells overexpressing Gtr1 or its constitutively active Gtr1^GTP^(Q65L) mutant based on higher TORC1 activity in *whi2* knockouts relative to wild type cells at 3 and 6 h after switching to low amino acids (Fig 5C and 5D). Consistent with this finding, co-expression of HA-Whi2 with Gtr1^GTP^(Q65L) suppresses the overzealous TORC1 activity (phospho-Rps6) induced by constitutively active Gtr1^GTP^(Q65L) and modestly suppresses the effects of expressed wild type Gtr1 (Fig 5E and 5F). Thus, because Whi2 can inhibit the effects of constitutively active GTP-bound Gtr1, any inhibitory effect of Whi2 on Gtr1 would presumably be by a mechanism different from the GAP activity of SEACIT (④ in Fig 3C), or alternatively, Whi2 could act in a pathway parallel to the Gtr complex (⑤ in Fig 3C). In the latter case, Whi2 could co-regulate TORC1 in conjunction with the Gtr1-Gtr2 GTPase complex.

To further distinguish a parallel Whi2 path (⑤ in Fig 3C) from a Gtr1-Gtr2-dependent path (④ in Fig 3C), we tested if Whi2 can act independently of these TORC1-activating RAG-like GTPases. We found that expression of HA-Whi2 potently suppresses TORC1 activity in both *∆gtr1* and *∆gtr2* (Fig 5G and 5H). This indicates that Whi2 does not require the Gtr complex to suppress TORC1 activity, and therefore is not upstream of the TORC1-activating RAG-like GTPases (④ in Fig 3C). Thus, Whi2 appears to function in a parallel independent path to communicate low amino acids signals to TORC1 (⑤ in Fig 3C).

In the event that Whi2 acts to suppress TORC1 in a pathway parallel to the GTPases, then deletion of both the TORC1 activator Gtr1 together with the TORC1 suppressor Whi2 would be expected to yield a neutralizing phenotype if both pathways are active simultaneously. Indeed, deletion of *WHI2* in *∆gtr1* reduces but does not abolish TORC1 activity (phospho-Rps6) after switching to low amino acid conditions (Fig 5I). Importantly, the double knockout *∆whi2∆gtr1* also exhibits an intermediate growth phenotype, consistent with the observed TORC1 activity (Fig 5J). Thus, Whi2 appears to reflect a novel alternative amino acid sensing pathway that signals amino acid insufficiency to TORC1 (⑤ in Fig 3C).

### Whi2 suppression of TORC1 activity requires Psr1 and Psr2

**Fig 6:**
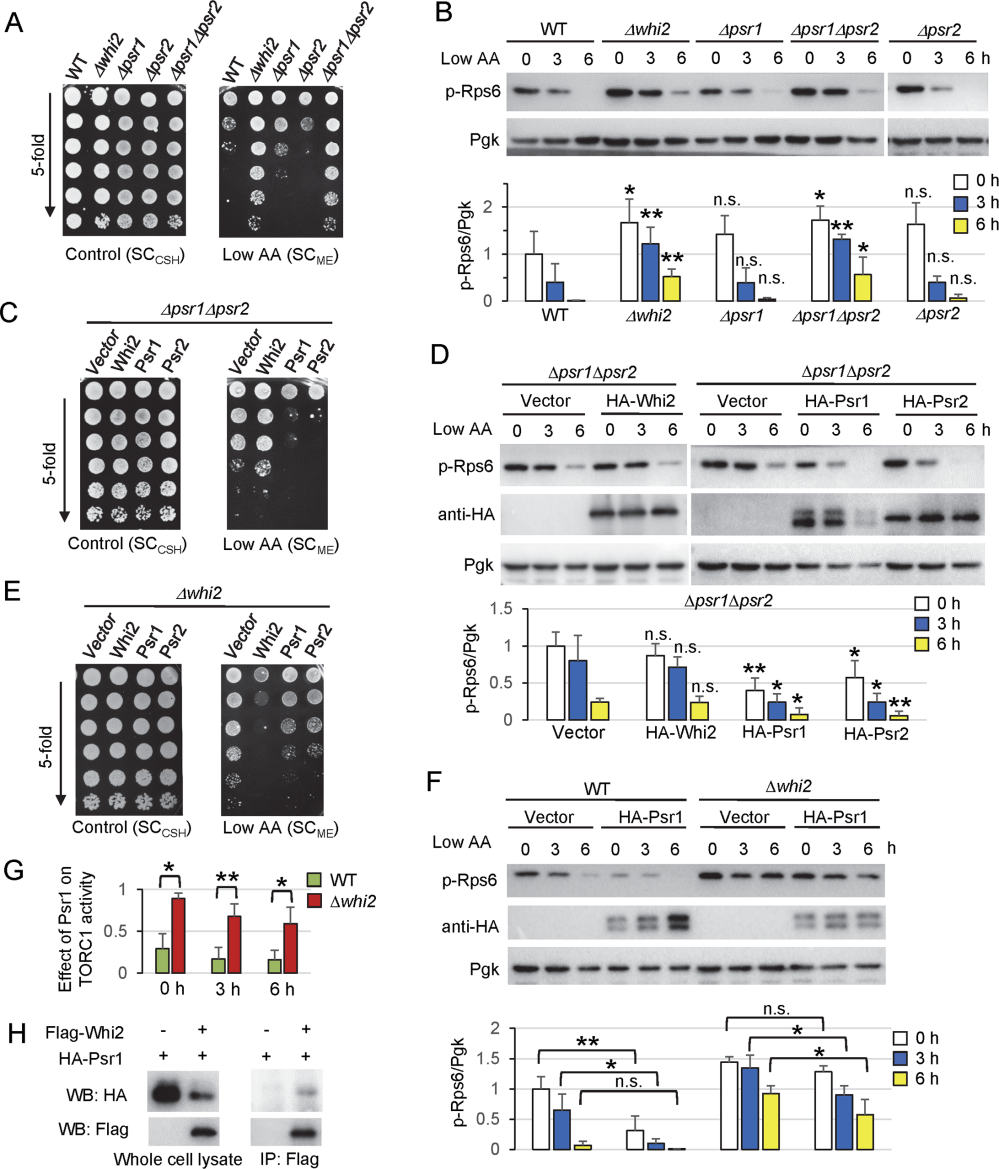
Whi2 suppression of TORC1 activity requires Psr1 and Psr2. **(**A, C, E) Growth of indicated yeast strains spotted in parallel in 5-fold serial dilutions on both control/high (SC_CSH_) to verify equal cell numbers and low amino acid plates (SC_ME_). Representative of ≥3 independent experiments is shown. (B) Immunoblot analysis for TORC1 activity assessed with anti-phospho-RpsS6 in indicated strains after switching from control/high (SC_CSH_) to low amino acid media (SC_ME_). Equal loading was achieved primarily by optical density-matched cultures and monitored with anti-Pgk. Corresponding bars below show quantification of TORC1 activity (the ratio of phospho-Rps6/Pgk). Values were normalized to the average value of WT at time zero, and presented in the bar graph as means ± SD (n = 4–5 independent experiments). **P < 0.01; *P < 0.05; n.s., not significant, compared to the respective WT control using a two-tailed T test. (D) Immunoblot analysis for TORC1 activity assessed with anti-phospho-RpsS6 in indicated strains after switching from control/high (SC_CSH_) to low amino acid media (SC_ME_). Equal loading was achieved primarily by optical density-matched cultures and monitored with anti-Pgk. Anti-HA was used to detect N-terminal HA-tagged Whi2, Psr1, and Psr2 expressed from the *PGK1* promoter in plasmids. Corresponding bars below show quantification of TORC1 activity (the ratio of phospho-Rps6/Pgk). Values were normalized to the average value of *Δpsr1Δpsr2* transformed with empty vector at time zero, and presented in the bar graph as means ± SD (n = 4 independent experiments). **P < 0.01; *P < 0.05; n.s., not significant, compared to the respective vector control using a two-tailed T test. (F) Immunoblot analysis was performed as for panel D for expressed Psr1 in wild type and *Δwhi2*. Corresponding bars below show quantification of TORC1 activity (the ratio of phospho-Rps6/Pgk). Values were normalized to the average value of WT transformed with empty vector at time zero, and presented in the bar graph as means ± SD (n = 4 independent experiments). **P < 0.01; *P < 0.05; n.s., not significant, compared to the respective vector control using a two-tailed T test. (G) Group data for the effect of Psr1 on TORC1 activity in WT versus *Δwhi2* from panel F. The ratios of TORC1 activity between indicated strains transformed with HA-Psr1 and empty vector were presented at each time point for 4 independent experiments. (H) Psr1 is co-immunoprecipitated with Whi2. HA-Psr1 was co-expressed with Flag-Whi2 or an empty vector in the WT (BY4741) strain. Anti-Flag immunoprecipitates were analyzed on immunoblots with anti-HA and anti-Flag.

Whi2 is reported to interact with the protein phosphatase Psr1 using recombinant proteins [10] and to physically interact with both Psr1 and Psr2 in several high-throughput screens [47–50]. We thus tested if Psr1 and its partially functionally redundant paralog Psr2 is involved in regulating TORC1 activity. Although single gene deletions of *PSR1* or *PSR2* had little or no overgrowth on low amino acids, the Δ*psr1*Δ*psr2* double knockout overgrew similarly to Δ*whi2* (Fig 6A). Consistent with this finding, Δ*psr1*Δ*psr2* also has sustained TORC1 activity after switching to low amino acid media, highly similar to Δ*whi2* tested in parallel (Fig 6B). These results suggest that Psr1 and Psr2 are required to suppress TORC1 activity under low amino acid conditions.

To determine if Psr1/Psr2 are also required for Whi2 to suppress TORC1, we overexpressed Whi2 in *Δpsr1Δpsr2*. Although expression of either Psr1 or Psr2 was sufficient to suppress overgrowth and TORC1 activity in *Δpsr1Δpsr2* under low amino acid conditions, expressed Whi2 had no effect (Fig 6C and 6D), indicating that Whi2 requires Psr1 or Psr2 to suppress TORC1 activity. Conversely, we found that the overexpressed enzymes Psr1 or Psr2 only weakly suppressed the overgrowth and the TORC1 activity of *Δwhi2* (Fig 6E). Importantly, expressed Psr1 reduced TORC1 activity in wild type cells much more efficiently than *Δwhi2* under low amino acid conditions, indicating that Psr1 also relies on Whi2 to fully suppress TORC1 (Fig 6F and 6G). Together, these reciprocal findings are consistent with Whi2 acting in a complex with Psr1 to suppress TORC1 activity. Supporting this model, we found that Whi2 and Psr1 can be co-immunoprecipitated (Fig 6H), consistent with the earlier study [10].

### Whi2 suppresses TORC1 activity independently of the PKA pathway

*WHI2*-deficiency is reported to cause inappropriate Ras-cAMP-PKA pathway activation [22]. However, when PKA activity was suppressed in *Δwhi2* by further deleting *TPK3*, which encodes a catalytic subunit of PKA [51], the *Δwhi2Δtpk3* double knockout was indistinguishable from *Δwhi2* for both overgrowth and TORC1 activity under low amino acid conditions (S4A and S4B Fig). As an alternative strategy, we overexpressed the high affinity cAMP phosphodiesterase Pde2, which inhibits PKA activity by hydrolyzing cAMP to AMP [51]. However, overexpressed Pde2 had no effect on the elevated growth or the TORC1 overactivity of *Δwhi2* (S4C and S4D Fig). Thus, at least under our low amino acid conditions, Whi2 suppresses TORC1 activity independently of the PKA pathway.

### Human KCTD11 suppresses mTORC1 activity in low amino acids

Although originally thought to be fungi-specific, Whi2 shares amino acid sequence similarity with a family of poorly characterized human proteins designated potassium channel tetramerization domain proteins (KCTDs) [1]. However, like fungal Whi2 proteins, human KCTD family proteins lack predicted channel domains and have very divergent C-termini, but share an N-terminal BTB domain subtype present in fungal Whi2. Among the 25 human KCTD family members [52], several have been linked to human cancers, including KCTD8 [53], KCTD12 [54], TNFAIP1 [3] and most notably KCTD11/Ren/KCASH1, a reported tumor suppressor in medulloblastoma, possibly by suppressing Hedgehog signaling [2, 55].

**Fig 7:**
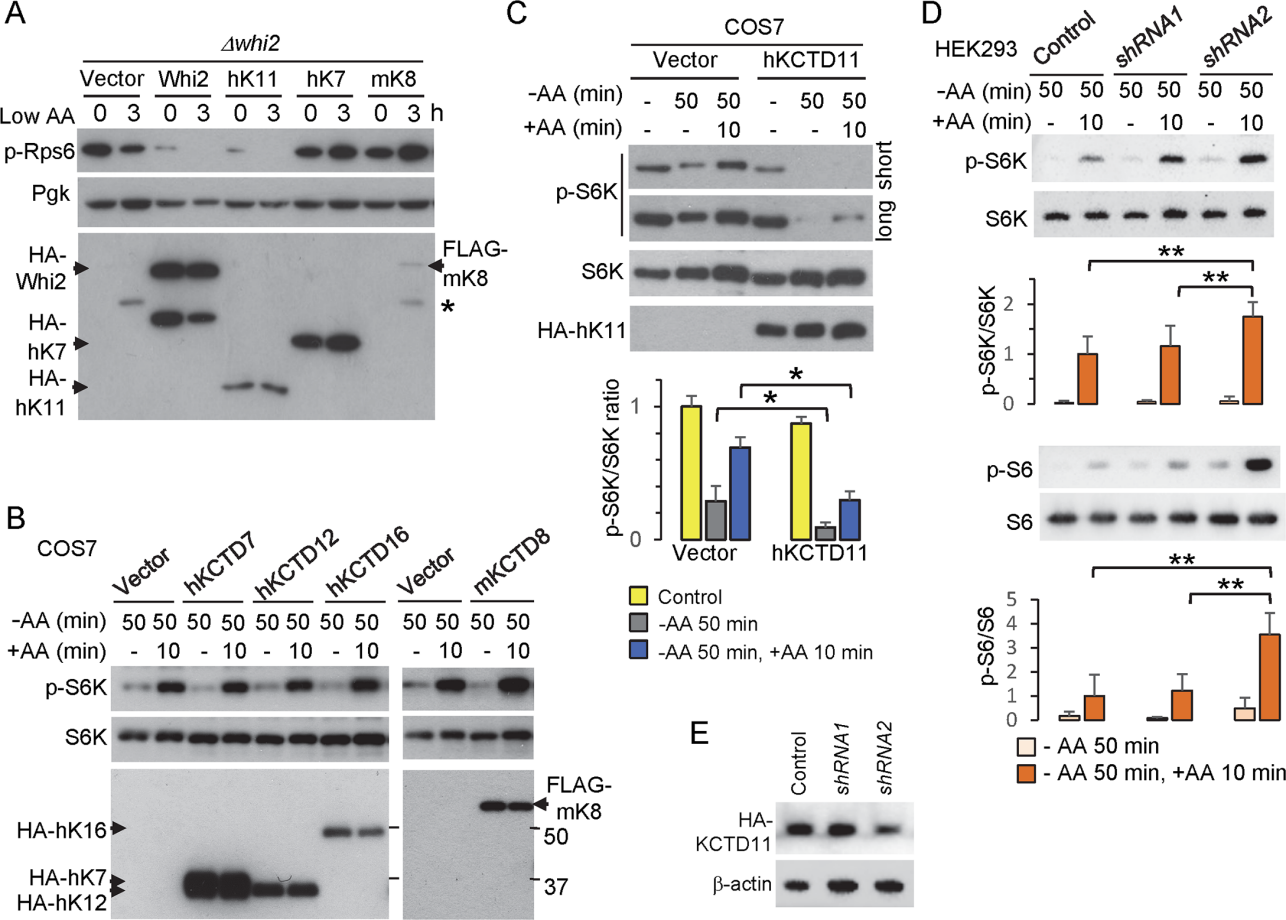
Mammalian KCTD11 is a negative regulator of mTORC1 in response to low amino acids. (A) Immunoblots for yeast TORC1 activity (anti-pS6) and Pgk loading control in *whi2* knockout yeast expressing N-terminal HA-tagged proteins, yeast Whi2, human KCTD11 (hK11), hKCTD7 (hK7), and mouse KCTD8 (mK8) proteins expressed from *PGK* promoter-driven plasmids before and after switching from control/high (SC_CSH_) to low amino acids (SC_ME_). Expressed proteins are detected with anti-HA antibody of the same lysates. *Presumed non-specific band. (B) The activity of mTORC1 in monkey kidney COS7 cells was assessed on immunoblots for phospho-S6K relative to total S6K in cells expressing HA-/FLAG-tagged mammalian KCTD proteins or empty vector harvested after 50 min of amino acid and serum deprivation with and without readdition of amino acids for 10 min. Representative of 3 independent experiments is shown. (C) As described in panel B for hKCTD11. Quantification of mTORC1 activity was determined as the ratio of phospho-S6K to total S6K for 3 independent experiments and presented as mean ± SD directly below corresponding lanes as labeled for immunoblots. Two-tailed t-test for cells transfected with HA-hKCTD11 versus empty vector after amino acid withdrawal, p = 0.047, and after readdition of amino acids, p = 0.003. (D) Endogenous KCTD11-dependent mTORC1 activity was analyzed in human HEK293 cells transfected with scrambled control and hKCTD11 shRNAs for 48 h followed by amino acid and serum deprivation for 50 min with and without readdition of amino acids for 10 min. Immunoblots for both phospho-S6K relative to total S6K (direct mTORC1 target) and for phospho-S6 relative to total S6 were quantified for 4–8 independent experiments and presented as mean ± SD directly below corresponding lanes as labeled for immunoblots. Two-tailed t-test comparing amino acid-treated samples for shRNA2 versus control shRNA (n = 8), p = 3.7 x 10^−4^ (p-S6K/S6K), p = 5.4 x 10^−5^ (p-S6/S6), and comparing shRNA2 versus ineffective shRNA1 (n = 4), p = 0.016 (p-S6K/S6K), p = 1.2 x 10^−3^ (p-S6/S6). (E) KCTD11 shRNAs were evaluated on immunoblots for their ability to suppress the expression of HA-tagged KCTD11 when co-transfected in human HEK293 cells for 24 h and grown under normal conditions.

To determine if mammalian KCTD proteins are also involved in regulating TORC1, we first tested their ability to suppress TORC1 activity in yeast, despite their limited overall sequence similarity with Whi2. N-terminal HA-tagged mammalian KCTD proteins KCTD7, KCTD8 and the extended version of KCTD11 containing the complete BTB domain [2] were expressed in yeast *Δwhi2*. Only KCTD11 suppressed TORC1 activity (phospho-Rps6) at baseline and further suppressed TORC1 activity in low amino acids similarly to HA-Whi2, while KCTD7 and KCTD8 had no effect or slightly increased TORC1 activity (Fig 7A). Human KCTD11 also mimicked yeast Whi2 by increasing expression of *prDAL80*-GFP, an alternative reporter for TORC1 inhibition [43] (S5A Fig), and also can suppress the overgrowth phenotype of *Δwhi2* on low amino acid plates similar to Whi2 (S5B Fig). Strikingly, expressed human KCTD11 can rescue the actin aggregation phenotype of *Δwhi2*, resulting in an actin-staining pattern similar to re-expressed Whi2 as previously reported [22] (S5C and S5D Fig). However, the specificity of KCTD11 in yeast is challenging to verify, therefore we tested KCTD11 in mammalian cells.

In mammalian cells (COS7), expression of KCTD7, KCTD8, KCTD12 and KCTD16 failed to suppress mTORC1 assessed by the phosphorylation status of mammalian S6 kinase (S6K) at T389, a direct target of mTORC1 [27], in amino acid-free medium (lacking serum, which contains amino acids) and after readdition of amino acids (Fig 7B). Again, only KCTD11 could suppress mTORC1 activity in mammalian cells after 50 min in amino acid-free medium and after readdition of amino acids (in the absence of serum) compared to control (Fig 7C). Importantly, endogenous KCTD11 also appears to negatively regulate mammalian TORC1 as transiently transfected shRNA2, and less effective shRNA1, consistently enhanced TORC1 activity based on the phosphorylation status of mTORC1 target S6K (Fig 7D upper), and its downstream target S6 (Fig 7D lower). Knockdown by shRNA2, in contrast to shRNA1, was confirmed in cells co-expressing human HA-KCTD11 (Fig 7E). These findings indicate that KCTD11 and yeast Whi2 may share an evolutionarily conserved role in suppressing TORC1 activity in response to reduced amino acid levels.

## Discussion

We found that *WHI2* is required to suppress TORC1 activity under low amino acid conditions. Because the GATOR1/SEACIT-Gtr1/2 pathway is the major regulator of TORC1 in response to both high and low amino acid levels [24, 26, 56], several strategies were used to probe the potential role for Whi2 in this pathway. Instead, we uncovered a parallel mechanism mediated by Whi2 that transmits low amino acid status to inhibit TORC1 activity independently of the SEACIT–Gtr GTPase axis (Figs 4 and 5). Thus, it appears that Whi2 and SEACIT-Gtr pathways are independent of each other, but when acting in concert, together they robustly suppress TORC1 activity (Fig 5). Thus, each could theoretically communicate non-overlapping information about nutrient status to TORC1. Yeast TORC1 can respond to specific amino acids including leucine [56], glutamine[57] and methionine [58], and mammalian mTORC1 was reported to respond to leucine [35], glutamine [59] and arginine [60]. The specific amino acids responsible for eliciting Whi2-dependent responses are not yet known, but leucine is the major difference between the two media used in this study [19].

The glucose-responsive Ras-cAMP-PKA pathway [61] appears not to be involved in Whi2-mediated suppression of TORC1 (S4 Fig), and Whi2 had no detectable role in suppressing TORC1 activity under low glucose conditions, at least in the presence of amino acids (Fig 3). This is in contrast to current opinion, although previous studies on low glucose conditions were performed in conjunction with nitrogen deprivation [9, 62].

The mechanism for Whi2-mediated TORC1 inhibition is not known, but requires the under-characterized phosphatases Psr1/Psr2 (Fig 6). Psr1 together with its binding partner Psr2 have been reported to interact with Whi2 in pull-down assays and/or high throughput screens [10, 47–50], confirmed for Psr1 by our co-IP with Whi2 (Fig 6H). Whi2 is suggested to activate the Psr1/Psr2 phosphatases, which are localized to the plasma membrane and have been implicated in mounting general stress responses by activating Msn2 [10]. Msn2 is reported to be suppressed when TORC1 is active [63], which is consistent with our model that Whi2 is an upstream negative regulator of TORC1. How or where Whi2-Psr1-Psr2 might regulate TORC1 directly or indirectly is not known.

How could the effect of Whi2–Psr1/Psr2 at the plasma membrane potentially connect to TORC1? One potential connection between Whi2 and TORC1 via Psr1/Psr2 is by controlling the activity of plasma membrane transporters. The ammonium transporter Mep2 was reported to be inactivated by Psr1/Psr2 [11]. In this model, Psr1/Psr2 removes the activating phosphorylation on Mep2 installed by Npr1 kinase when nutrients are low and Npr1 is not inactivated by TORC1 [11]. Consistent with their findings, an alternative possibility is that Whi2-Psr1/Psr2 acts upstream of TORC1 to suppress nutrient uptake.

Another potential mechanism connects plasma membrane amino acid transporters to TORC1. The general amino acid permease Gap1 and the arginine-specific transporter Can1 appear to function as a type of amino acid sensor by fluxing protons during amino acid uptake [64]. This proton flux triggers compensatory export of protons by the plasma membrane H^+^-ATPase Pma1. By an unknown proton-activated Pma1-dependent signaling mechanism, Pma1 activates TORC1[64]. However, any role for Whi2 in this pathway is not known and unlike Whi2, the ability of Pma1 to regulate TORC1 is at least partially dependent on the GATOR1/SEACIT-Gtr pathway [64]. Furthermore, the proton-Pma1 pathway to activate TORC1 was postulated only to respond to nutrient availability and not to be involved in the suppression of TORC1 activity in low nutrients [64].

The requirement for Whi2 to suppress TORC1 in low amino acid conditions implies that *Δwhi2* cells will be defective for autophagy induction due to sustained TORC1 activity, consistent with a recent study [5]. Mendl et al. reported that Whi2 is required for the degradation of mitochondria via autophagy (mitophagy) induced by high concentrations of rapamycin (1 μM) for 7.5–24 hours [65]. However, a conflicting report from Klionsky and colleagues concluded that Whi2 is not required for mitophagy when stationary cells grown in lactate are switched to nitrogen starvation medium plus 2% glucose to induce mitophagy [66].

Whi2-dependent amino acid sensing could be important for fungal pathogenesis. Down regulation of TORC1 in pathogenic strains of yeast such as *Candida albicans* and *Candida glabrata* is suggested to play a role in the persister state [67]. Consistent with our finding that Whi2 suppresses TORC1, the Whi2 ortholog in the fungal pathogen *Colletotrichum orbiculare* was recently suggested to have a role in plant pathogenesis by inhibiting TOR signaling [68].

The evidence presented for Whi2 being a negative regulator of TORC1 is supported by several different readouts for TORC1 activity. We detected TORC1 activity using a specific antibody for highly conserved phosphorylation sites in the ribosomal S6 proteins of both yeast (Rsp6 Ser232/Ser233) and mammals (Ser235/Ser236), which are known to be phosphorylated downstream of mTORC1 in response to amino acids in mammals [35, 36]. This antibody permits quantification of yeast TORC1 activity by monitoring an endogenous substrate without reporters or gel-shift assays. TORC1-dependent phosphorylation of yeast Rps6 is further supported by rapid dephosphorylation at Ser232/233 upon rapamycin treatment (Fig 3). Rps6 phosphorylation is regulated by RAG-like GTPases Gtr1-Gtr2 in wild type cells (Fig 5), verifying the utility of this strategy. Two alternative TORC1 activity assays (*DAL80* expression and Npr1 phosphorylation), further confirmed that Whi2 is required to suppress TORC1 activity under low amino acid conditions (S3 Fig).

It is puzzling that *WHI2* was not identified in several screens for regulators of low amino acid-sensing in the TORC1 pathway [43, 69, 70]. Possibly the death-sensitivity of *Δwhi2* to multiple stimuli [1, 10, 19] results in loss of *whi2* mutants during screening under more harsh conditions. In contrast to other reports [9, 41], we found that yeast Whi2 is not required to suppress TORC1 activity in response to low glucose in the presence of amino acids (Fig 2). *WHI2* was identified in one other screen for suppressors of TORC1 [41]. However it was concluded that the apparent suppressive effects of *WHI2* on TORC1 activity following glucose or nitrogen depletion was more likely due to indirect consequences of impaired cell cycle arrest by *whi2* mutants in a chemostat environment [41]. Conversely, we and others observed spontaneous *whi2* mutations in specific knockout strains that appear to arise in part due to the loss of specific knockout gene functions [1, 19, 71]. Perhaps the selection for *whi2* mutations is related to the role of Whi2 in regulating TORC1 as amino acids become depleted during normal culturing.

In further support of a specific role for Whi2 in sensing low amino acids, the protein level of Whi2 rapidly increases upon shifting to low amino acid conditions, followed by suppression of TORC1 activity (Fig 2D). In contrast, glucose limitation has the opposite effect, reducing Whi2 protein levels. Interestingly, we also observed that Iml1 (component of SEACIT) levels increase following switch to low amino acids (Fig 4A). The fact that induction or stabilization of Whi2 and Iml1 protein levels is an early event in response to low amino acids suggests that Whi2 and Iml1 are both effectors of an upstream signal.

Given the high prevalence of secondary mutations in knockout strains [1], we performed tetrad analysis on the *Δwhi2*, *Δnpr2*, *Δnpr3*, *Δgtr1* and *Δgtr2* strains used in this study, which verified that the phenotypes described here are appropriately attributed. However, the *Δgtr2* strain in the BY4741 YKO collection is a mix of two prominent phenotypes with dramatically different sensitivities to stress [1]. Although the yet unidentified single secondary gene mutation responsible for stress sensitivity of *Δgtr2* did not affect amino acid sensing [1], a substrain lacking this secondary mutation was used for these studies. No secondary mutations that contribute to the growth phenotypes were identified in multiple colonies tested of *Δwhi2*, *Δnpr2*, *Δnpr3*, *Δgtr1* and *Δgtr2* strains used in this study. In contrast, the tetrads generated from knockouts strains for Ego1 and Ego3, which anchor Gtr1-Gtr2 to membranes [56], exhibited complex genetics/phenotypes and therefore were not further studied. It remains possible that the SEACIT-Gtr and the Whi2 pathways converge near or within the TORC1 protein complex.

Other Gtr/RAG-independent mechanisms involving different GTPases have been reported in both yeast and mammals to activate TORC1 in response to amino acid abundance. The mammalian Rab family GTPase Rab1A and its yeast homolog Ypt1, an essential gene, appear to activate TORC1 independently of RAG/Gtr GTPases [72]. However, it is not known if yet unidentified negative regulators of the Ypt1/RAB1A GTPases might transmit low amino acid status to suppress TORC1, analogous to Whi2 or SEACIT/GATOR1. Another RAG GTPase-independent mechanism in *Drosophila* and mammalian cells requires the ARF1 GTPase for acute TORC1 activation induced by glutamine [59]. This ARF1-dependent mechanism has not been demonstrated in yeast. To the contrary, we identified *Δarf1* in the same screen that identified *Δnpr2 and Δnpr 3* [1], implying that yeast Arf1 may be a negative rather than positive regulator of TORC1. However, it is not known if this phenotype is due to deletion of *ARF1* or to a secondary mutation. Yeast Arf1 also activates the Ras-cAMP-PKA pathway particularly in response to glucose [61]. However, our findings indicate that *whi2*-deficient cells respond normally by suppressing TORC1 activity and cell growth in glucose-free conditions, and that the sustained TORC1 activity and cell growth are not dependent on the PKA pathway (S4 Fig).

These Gtr/RAG-independent amino acid-sensing paths involving Ypt1/RAB1A and ARF1 could potentially be connected. Unlike the RAG/Gtr complexes, which localize to the lysosome/vacuole membrane [73], RAB1A and ARF1 are both predominantly localized on Golgi membranes, supporting a model for TORC1 to sense amino acids in different subcellular regions [74]. Whi2 physically interacts with the phosphatase Psr1/2, which localizes to the plasma membrane [10], and endogenous Whi2 fluorescently tagged (either N- or C-terminus) has also been shown to localize to the cell periphery [75]. Our study shows that Whi2 inhibits TORC1 activity through Psr1 and Psr2, raising the possibility of Whi2 extending amino acid signaling to additional subcellular sites. Importantly, Whi2 contributes to the suppression of TORC1 activity that occurs in the absence of Gtr1 (Fig 5G–5I).

We provide the first evidence that an amino acid-sensing function of yeast Whi2 is conserved in a mammalian KCTD family protein, KCTD11. The evolutionary histories of Whi2 and KCTD family proteins have not yet been studied in detail. At this stage therefore, no reliable homology can be inferred beyond the SKP1/BTB/POZ domain shared between yeast and human protein types (note that subsets of the human KCTD family also have sequence similarities concentrated only in their SKP1/BTB/POZ domain). Yet, it is remarkable that KCTD11 can functionally substitute for yeast Whi2 to suppress TORC1 activity and cell growth under low amino acid conditions, and to rescue the actin filament aggregation phenotype of *Δwhi2*. Importantly, both expressed and endogenous KCTD11 also negatively regulates mTORC1 activity under amino acid depletion conditions in mammalian cells, confirming the conserved function of TORC1 suppression. It was somewhat unexpected that KCTD7 did not affect mTORC1 activity given that fibroblasts from EPM3 patients with bi-allelic *KCTD7* mutations have defective autophagy responses [5]. Biallelic *KCTD7* mutations define the diagnosis of EPM3 (progressive myoclonic epilepsy-3), a severe neurodegenerative disorder with onset in early childhood [4, 76]. It remains possible that KCTD7 and other family members modulate this pathway in other conditions or cell types. However, KCTD family proteins have received little attention and remain poorly characterized despite their disease associations. KCTD11 was reported to suppress the Hedgehog signaling pathway in medulloblastoma [2], and crosstalk between the Hedgehog and PI3K/AKT/mTORC1 pathways via Gli1 activation has been reported to occur in several types of cancer models including medulloblastoma, prostate cancer and breast cancer cell lines [77]. KCTD11 also has been implicated as a tumor suppressor in several other cancers including prostate adenocarcinoma [18], and hepatocellular carcinoma [17], although its role in cancer remains to be confirmed.

In summary, we investigated the possibility that Whi2 is a new upstream negative regulator of TORC1. Indeed, *whi2*-deletion strains have sustained TORC1 activity (phospho-Rps6) following a switch to medium with lower amino acids. Both the overgrowth and sustained phospho-Rps6 levels are dependent on TORC1 as both are blocked by low concentrations of rapamycin. However, we found that Whi2 suppresses TORC1 activity independently of the RAG-like Gtr complex conserved in yeast, but through protein phosphatases Psr1 and Psr2, implying a novel mechanism. Despite amino acid sequence divergence from yeast Whi2, the human protein KCTD11, but not other KCTD family members tested under our conditions, suppress TORC1 activity in yeast. More importantly, endogenous KCTD11 suppresses mTORC1 activity in mammalian cells as KCTD11 depletion leads to higher mTORC1 activity. Thus, it is conceivable that failure to suppress mTORC1 in tumors lacking functional KCTD11 may be an important contributor to its proposed role in tumorigenesis [2, 17, 18].

## Materials and methods

### Yeast strains and plasmids

The *Saccharomyces cerevisiae* strains and the yeast and mammalian expression plasmids used in this study are listed for each figure (Tables 1 and 2, respectively).

**Table 1:** Yeast strains used in this study.

Strain	Genotype	Source	Figure
WT BY4741	*MATa his3 leu2 ura3 met15*	[78]	1; 2A, 2B; 3; 5C–5J; 6A, 6B, 6F–6H; S3–S5
WT BY4742	*MATα his3 leu2 ura3 lys1*	[78]	S2
Δ*fis1-d1*	[BY4741] *fis1*::*KanMX4 whi2-1*	[19]	1A–1D; 2B
Δ*whi2-1*	[BY4741] *whi2*::*KanMX4*	[1, 78]	1A–1D, 1G; 2A, 2B; 3; 4A–4C; 5A–5D, 5I, 5J; 6A, 6B, 6E–6G; 7A; S2–S5
Δ*npr2-2*	[BY4741] *npr2*::*kanMX4*	[1, 78]	3; 4D; S2; S3
Δ*npr3-1*	[BY4741] *npr3*::*kanMX4*	[1, 78]	3; 4E; S2; S3A
Δ*gtr1-1*	[BY4741] *gtr1*::*kanMX4*	[1, 78]	5G–5J
Δ*gtr2-2*	[BY4741] *gtr2*::*kanMX4*	[1, 78]	5G
Δ*whi2*Δ*gtr1*	[BY4741] *gtr1*::*kanMX4 whi2*::*HIS3*	This study	5I and 5J
Whi2-TAP	[BY4741] Whi2-TAP	[42]	2C and 2D
Δ*psr1*	[BY4742] *psr1*::*kanMX4*	[78]	6A and 6B
Δ*psr2*	[BY4742] *psr2*::*kanMX4*	[78]	6A and 6B
Δ*psr1*Δ*psr2*	[BY4742] *psr1*::*kanMX4 psr2*::*HIS3*	This study	6A–6D
Δ*tpk3*	[BY4742] *tpk3*::*kanMX4*	[78]	S4A and S4B
Δ*whi2*Δ*tpk3*	[BY4742] *whi2*::*kanMX4 tpk3*::*HIS3*	This study	S4A and S4B

**Table 2:** Plasmids used in this study.

Plasmid	Description	Source	Figure
Ycplac33	CEN, *URA3*	[79]	
WCC6	[Ycplac33] *prWHI2-WHI2*	[19]	1D
BQ23	2μ, *URA3*, *prPGK1*	This study	1G; 4; 5G, 5H; 6C–6F; 7A
TXC19	[BQ23] *prPGK1-HA-WHI2*	This study	1G; 4; 5G, 5H; 6C–6F; 7A
CXH4	[BQ23] *prPGK1-HA-NPR2*	This study	4B–4D
CXH5	[BQ23] *prPGK1-HA-NPR3*	This study	4B, 4C, 4E
RS416	CEN, *URA3*	[80]	
p2285	[pRS416] *prADH1-IML1-HIS6-TEV-ProtA*	[24]	4A and 4C
BQ8	2μ, *URA3*, *prPGK1*	This study	5A
Gtr1	[BQ8] *prPGK1-GTR1*	This study	5A–5F
Gtr1^GDP^	[BQ8] *prPGK1-GTR1-S20L*	This study	5A and 5B
Gtr1^GTP^	[BQ8] *prPGK1-GTR1-Q65L*	This study	5A–5F
RS313	CEN, *HIS3*	[81]	5E and 5F
ZY1	[pRS313] *prPGK1-HA-WHI2*	This study	5E and 5F
WGQ1	[BQ23] *prPGK1-HA-PSR1*	This study	6C–6H
WGQ2	[BQ23] *prPGK1-HA-PSR2*	This study	6C–6E
ZMJ11	[pRS313] *prPGK1-3*x*FLAG-WHI2*	This study	6H
TXC20	[BQ23] *prPGK1-HA-hKCTD7*	This study	7A
TXC21	[BQ23] *prPGK1-HA-mKCTD8*	This study	7A
TXC33	[BQ23] *prPGK1-HA-hKCTD11*	This study	7A
DB59	*prSV40*, *N-HA*	This study	7B and 7C
HA-hKCTD7	[DB59] *prSV40-HA-hKCTD7*	This study	7B
HA-hKCTD12	[DB59] *prSV40-HA-hKCTD12*	This study	7B
HA-hKCTD16	[DB59] *prSV40-HA-hKCTD16*	This study	7B
FLAG-mKCTD8	[pCI] *prCMV-FLAG-mKctd8*	[15]	7B
HA-hKCTD11	[DB59] *prSV40-HA-hKCTD11*	This study	7C, 7E; S5A and S5B
WGQ4	[Ycplac33] *prDAL80-GFP*	This study	S3A
AS103	[YEplac195] *prNPR1-HA-NPR1*	[45]	S3B
ZMJ2	[pRS313] *prPGK1-HA-PDE2*	This study	S4C and S4D
WGQ5	[pRS313] *prDAL80-GFP*	This study	S5A

### Yeast media and growth assays on plates

Yeast cultures were grown for 48 h in liquid YPD (2% peptone, 1% yeast extract, and 2% dextrose) or SC_CSH_ [19] (0.67% yeast nitrogen base w/o amino acids, 0.2% CSH amino acid mix, 2% glucose) minus uracil or/and histidine for strains transformed with *URA3* or/and *HIS3* plasmids. Saturated cultures were washed and serially diluted fivefold in sterile ddH_2_O, and 5 μL of each dilution were spotted onto solid SC_CSH_ and SC_ME_ [19] (0.67% yeast nitrogen base w/o amino acids, 0.124% ME amino acid mix, 2% glucose) with/without rapamycin, or SC_CSH_ containing indicated concentration of glucose, and incubated at 30 ^o^C for two to three days.

### TORC1 activity by phospho-Rps6 immunoblot analysis in yeast

Yeast strains were grown overnight in liquid SC_CSH_, and refed for 1 h in fresh SC_CSH_ medium at 1 OD/mL to allow diauxic phase cells to recover from nutrient deprivation overnight. Yeast cultures were washed once and equal cell numbers were resuspended in low amino acid medium SC_ME_. Lysates were prepared as reported [20], separated on 12% SDS-PAGE gels and analyzed on immunoblots with antibodies against phosphorylated Rps6 (mammalian phospho-S235/236 S6 antibody, Cell Signaling Technology, 1:1000), yeast Pgk (Abcam, 1:1000), HA-epitope and β-actin (Santa Cruz Biotechnology, 1:1000), followed by HRP-conjugated anti-rabbit and anti-mouse secondary antibodies (GE Healthcare, 1:20,000). TORC1 activity was quantified as a ratio of the intensity of phosphorylated Rps6 relative to loading control for each sample in ImageJ.

### TORC1 activity by *prDAL80*-GFP expression in yeast

Yeast strains transformed with *prDAL80*-GFP plasmid were cultured and lysed as described in the above section. Cell lysates were resolved on 12% SDS-PAGE gels and analyzed on immunoblots with antibody against GFP (Santa Cruz Biotechnology, 1:1000) and yeast Pgk (Abcam, 1:1000), followed by HRP-conjugated anti-rabbit and anti-mouse secondary antibodies (GE Healthcare, 1:20,000). TORC1 activity was quantified as a ratio of the intensity of GFP relative to loading control for each sample in ImageJ.

### TORC1 activity by Npr1 phosphorylation in yeast

Yeast strains transformed with *HA*-*NPR1* plasmid were cultured and lysed as described in the above section. Cell lysates were resolved on 7.5% SDS-PAGE gels and analyzed on immunoblots with antibody against HA-epitope (Santa Cruz Biotechnology, 1:1000) and yeast Pgk (Abcam, 1:1000), followed by HRP-conjugated anti-rabbit and anti-mouse secondary antibodies (GE Healthcare, 1:20,000). TORC1 activity was measured by the shift of the HA-Npr1 band.

### Actin staining in yeast

Yeast strains were grown in liquid SC_CSH_ for 24 h before staining F-actin with Rhodamine-phalloidin as reported [82]. Cells were viewed with a Nikon TE-2000 fluorescence microscope.

### Cloning of human KCTD family members

Human KCTD11 cDNA consisting of the KCTD11 ORF (NM_001002914.2) plus 120 5’ in-frame nucleotides required to complete the conserved BTB domain [83] were PCR amplified from HEK293 cells (5’ primer GGGAGATCGAAGATCTAAAATTTCTCCTCCTCCTGTGCCCTCTTCG, 3’ primer CTTGGTGACCAGATCTTCAGTGCCGGACAAAGCGCAGAGAC), and subcloned into the BglII site of pSG5-based vector pDB59 containing a Kozak sequence and N-terminal HA-tag and verified by Sanger sequencing. cDNA of human KCTD7 (NM_153033.4), KCTD12 (NM_138444.3), and KCTD16 (NM_020768.3) were cloned similarly into the BglII site of pDB59 vector. Primers used to amplify each gene are listed below. KCTD7: 5’ primer AGCTAGATCTATGGTGGTAGTCACGGGG, 3’ primer AGCTAGATCTTCACCACCATGTGATCTTGAA; KCTD12: 5’ primer AGCTAAGCTTGGATGGCTCTGGCGGACAG, 3’ primer AGCTAAGCTTTCACTCCCTGCAGAAGACG; KCTD16: 5’ primer AGCTAAGCTTATGGCTCTGAGTGGAAACTGTAG, 3’ primer AGCTAAGCTTTTATAGATGATACTTCCTTAAAAGTTCAGATTGCCAA.

### Immunoblot analysis for mTORC1 activity in mammalian cells

Expressed KCTD family proteins were tested in monkey COS-7 cells (from ATCC) cultured in DMEM containing 10% fetal bovine serum (FBS) and Penicillin/Streptomycin antibiotics (Hyclone), and endogenous KCTD11 was evaluated in human embryonic kidney 293 cells (from ATCC) cultured in DMEM plus 10% FBS and P/S. Approximately 2x10^4^ cells were seeded into 12-well plates with 1 mL media, and transfected 24 h later with 0.75 μg DNA for 24 h (protein expression plasmids) or for 48 h (shRNA plasmids) using Lipofectamine 2000 according to manufacturer’s instructions. Plasmids containing shRNAs targeting KCTD11 from GeneCopia ([psi-LVRH1GP] prH1-shRNA-EGFP) have the following target sequences:

Human hKCTD11 shRNA1: cttccggcacatcctcaat
Human hKCTD11 shRNA2: aggctgacttctaccagat

All transfected cells were rinsed once with amino acid-free RPMI (US Biological), incubated in 1 mL amino acid-free RPMI for 50 min, and stimulated by adding 20 μL of a 50× amino acid mixture (RPMI 1640 Amino Acids Solution, Sigma) for 10 min. Cell lysate were prepared in lysis buffer (62.5 mM Tris-HCl, pH = 6.8, 2% w/v SDS, 10% glycerol, 0.01% w/v bromophenol blue), separated on 12% gels by PAGE and analyzed on immunoblots using antibodies against phospho-S235/235 S6, S6, phospho-T389 S6K, S6K, (Cell Signaling Technology) at 1:1000 dilution, followed by HRP-conjugated anti-rabbit secondary antibodies (GE Healthcare, 1:20,000). To test the efficiency of KCTD11 shRNA knockdowns, HEK293 cells were co-transfected with HA-KCTD11 at a 1:9 ratio of hHA-KCTD11:shRNA (0.75 μg total DNA per well) and harvested for immunoblot analysis after 24 h. mTORC1 activity was quantified as a ratio of the intensity of phosphorylated S6K/S6 relative to total S6K/S6 for each sample in ImageJ. The values were normalized to the average value of the control.

## Supporting information

S1 Fig Fungal Whi2 proteins share significant amino acid sequence similarity to mammalian KCTD family proteins in the SKP1/BTB/POZ domain. (TIFF)

S2 Fig Overgrowth on low amino acids genetically segregates to the knockout locus in *Δwhi2*, *Δnpr2* and *Δnpr3* and not to secondary mutations. (TIFF)

S3 Fig Alternative TORC1 activity assays confirm that *WHI2* is required to suppress TORC1 activity in low amino acids. (TIFF)

S4 Fig Whi2 suppresses TORC1 activity independently of the PKA pathway. (TIFF)

S5 Fig KCTD11 can rescue the overgrowth, TORC1 overactivity and actin aggregation in *Δwhi2* under low amino acid conditions. (TIFF)
