# Supplementary figures and images for: Whi2 is a conserved negative regulator of TORC1 in response to low amino acids
Source: PLoS Genet. 2018 Aug 24;14(8):e1007592. doi: 10.1371/journal.pgen.1007592 (PMC6126876; doi:10.1371/journal.pgen.1007592)

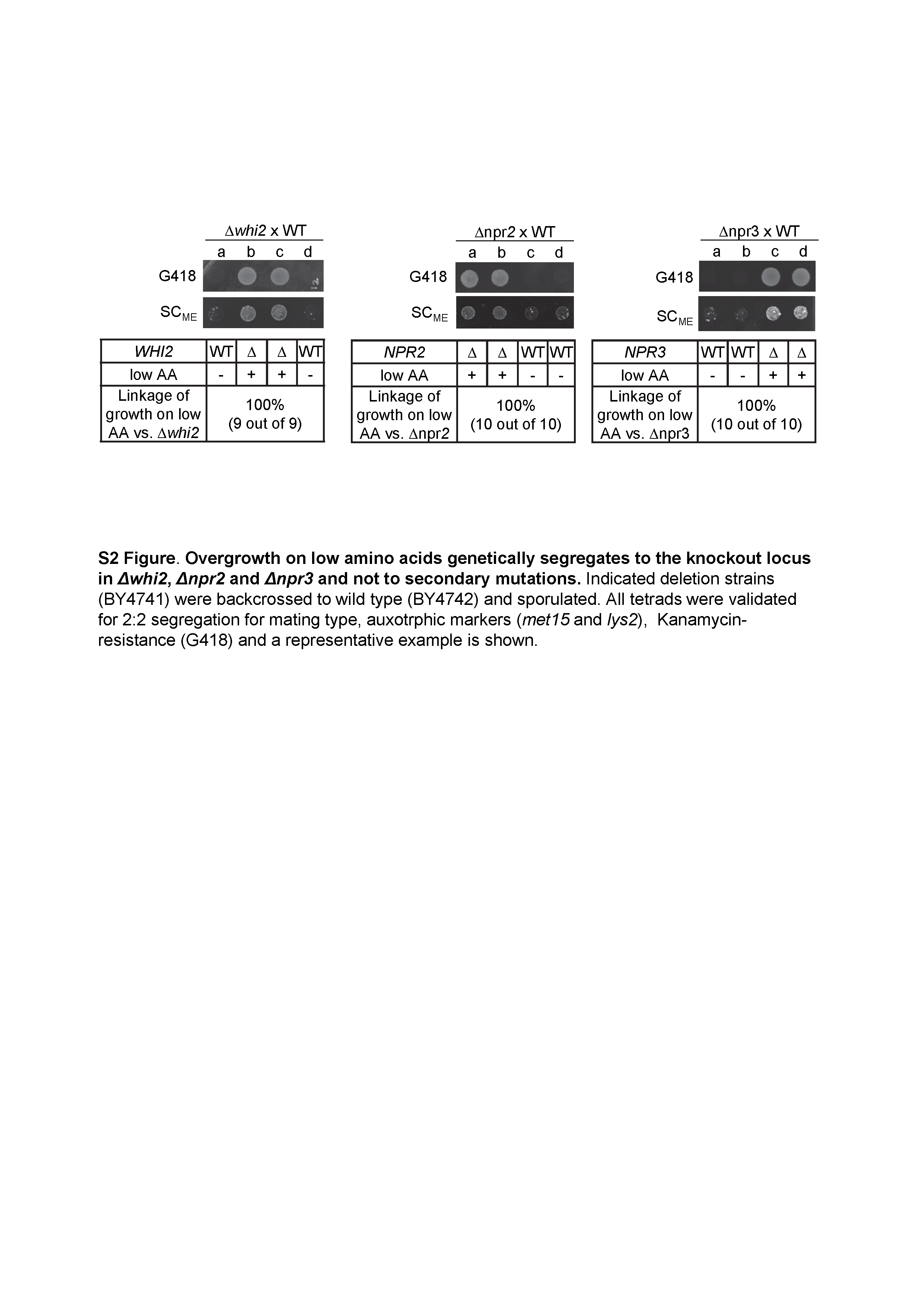

Supplement: S2 Fig — (TIFF) [file pgen.1007592.s002.tiff]

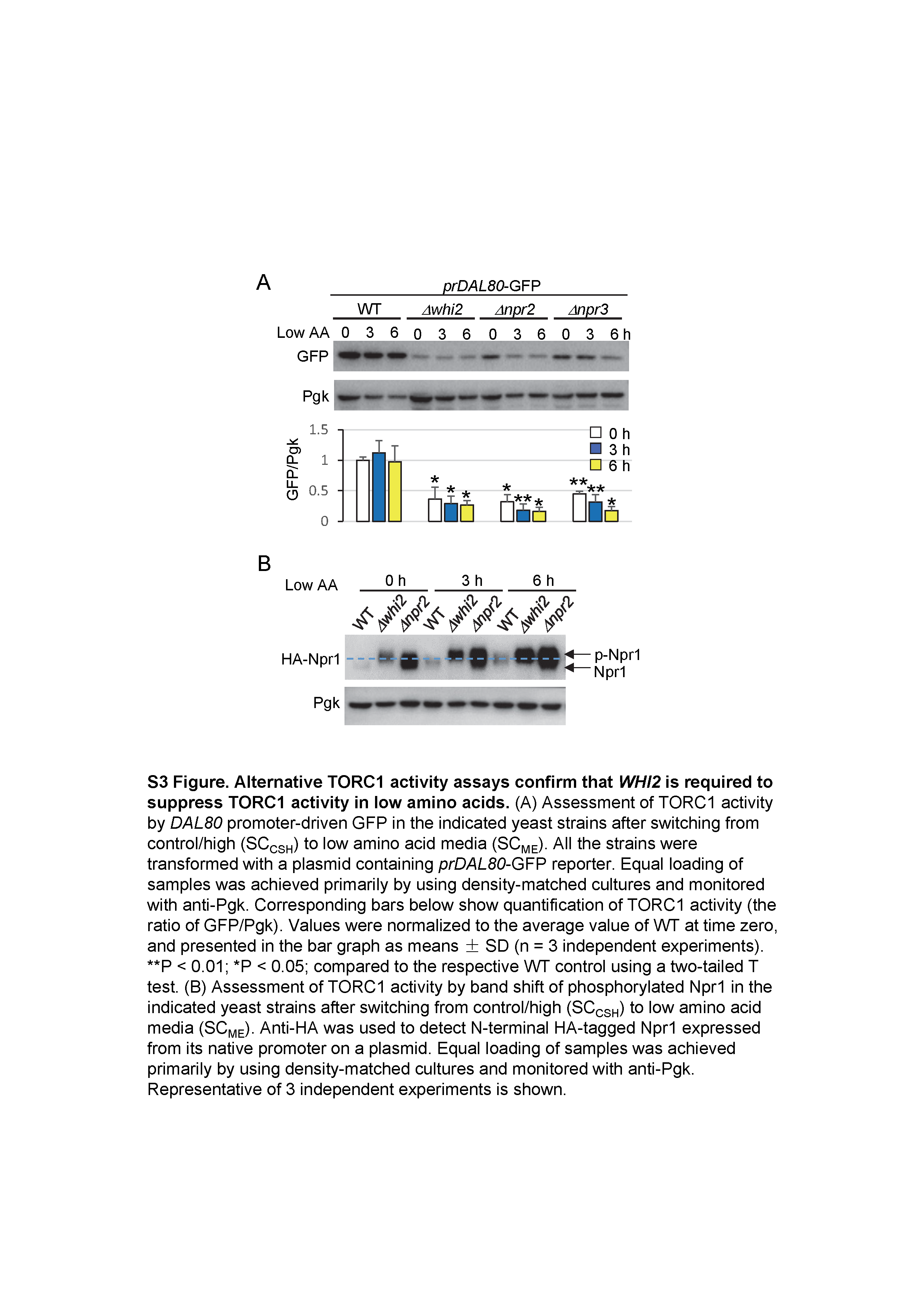

Supplement: S3 Fig — (TIFF) [file pgen.1007592.s003.tiff]

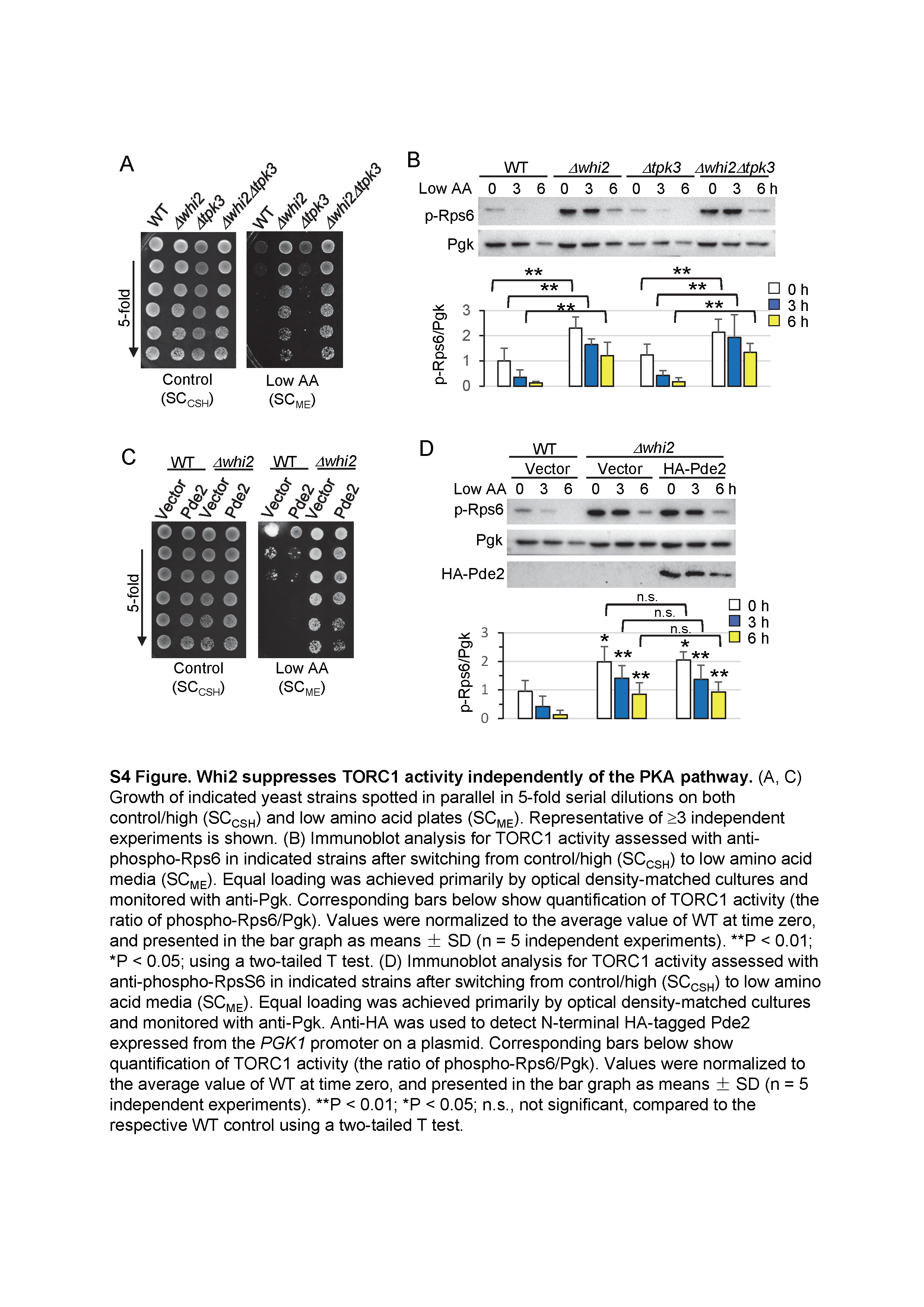

Supplement: S4 Fig — (TIFF) [file pgen.1007592.s004.tiff]

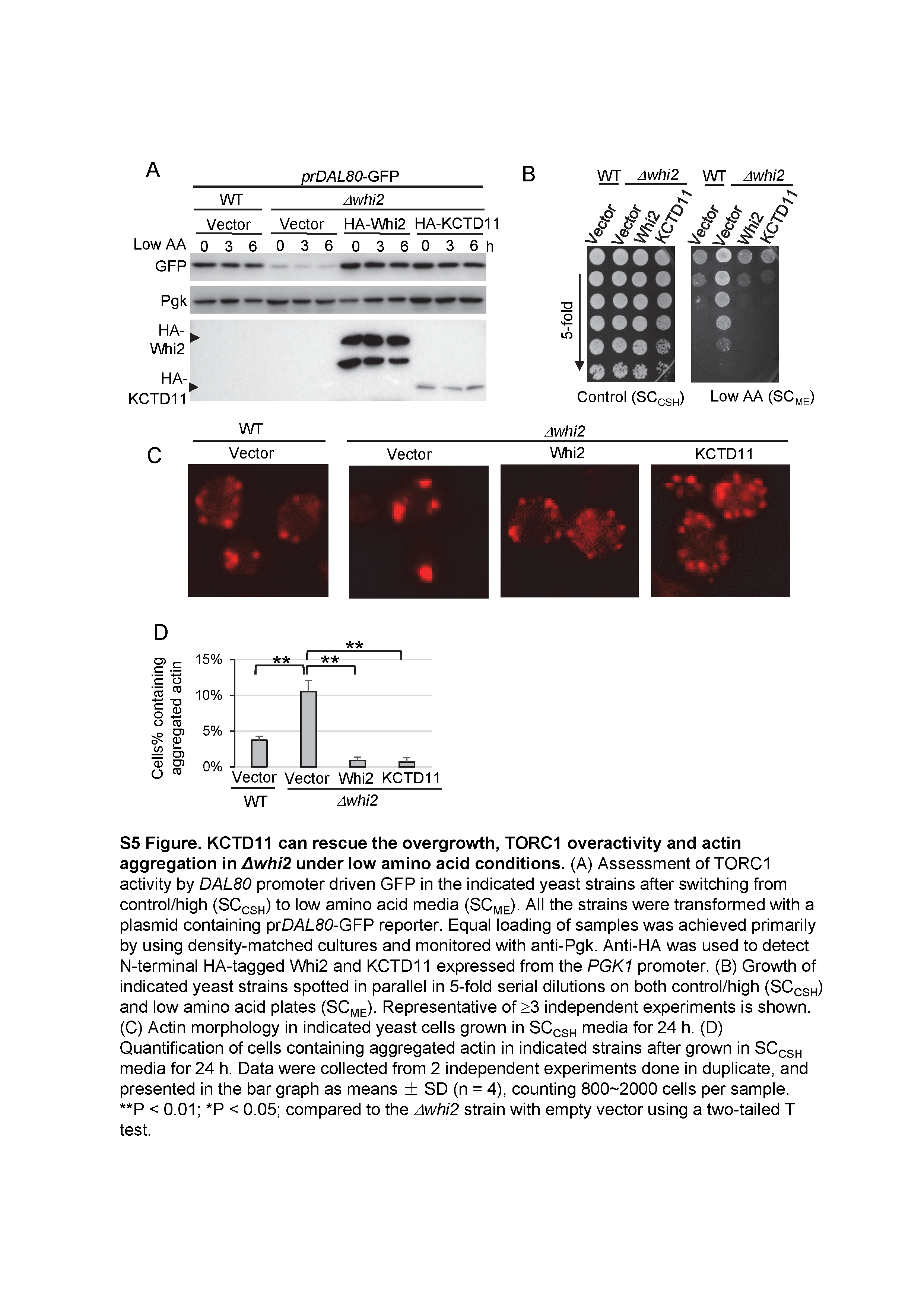

Supplement: S5 Fig — (TIFF) [file pgen.1007592.s005.tiff]
